# Pullulanase and Starch Synthase III Are Associated with Formation of Vitreous Endosperm in Quality Protein Maize

**DOI:** 10.1371/journal.pone.0130856

**Published:** 2015-06-26

**Authors:** Hao Wu, Kasi Clay, Stephanie S. Thompson, Tracie A. Hennen-Bierwagen, Bethany J. Andrews, Bernd Zechmann, Bryan C. Gibbon

**Affiliations:** 1 Department of Biology, Baylor University, Waco, Texas, 76798, United States of America; 2 Iowa State University, Department of Biochemistry, Biophysics, and Molecular Biology, Ames, Iowa, 50011, United States of America; 3 Texas A&M University, Department of Soil and Crop Sciences, College Station, Texas, 77843, United States of America; 4 Center for Microscopy and Imaging, Baylor University, Waco, Texas, 76798, United States of America; 5 Department of Biological Sciences, Florida A&M University, Tallahassee, Florida, 32307, United States of America; Murdoch University, AUSTRALIA

## Abstract

The *opaque-2* (*o2*) mutation of maize increases lysine content, but the low seed density and soft texture of this type of mutant are undesirable. Lines with modifiers of the soft kernel phenotype (*mo2*) called “Quality Protein Maize” (QPM) have high lysine and kernel phenotypes similar to normal maize. Prior research indicated that the formation of vitreous endosperm in QPM might involve changes in starch granule structure. In this study, we focused on analysis of two starch biosynthetic enzymes that may influence kernel vitreousness. Analysis of recombinant inbred lines derived from a cross of W64A*o2* and K0326Y revealed that pullulanase activity had significant positive correlation with kernel vitreousness. We also found that decreased Starch Synthase III abundance may decrease the pullulanase activity and average glucan chain length given the same Zpu1 genotype. Therefore, Starch Synthase III could indirectly influence the kernel vitreousness by affecting pullulanase activity and coordinating with pullulanase to alter the glucan chain length distribution of amylopectin, resulting in different starch structural properties. The glucan chain length distribution had strong positive correlation with the polydispersity index of glucan chains, which was positively associated with the kernel vitreousness based on nonlinear regression analysis. Therefore, we propose that pullulanase and Starch Synthase III are two important factors responsible for the formation of the vitreous phenotype of QPM endosperms.

## Introduction

Normal maize lines cannot provide a nutritionally balanced source of protein because of the deficiency of lysine [1]. Most cereal grains contain 1.5%-2% lysine, which is less than a half of the amount required for human nutrition [2]. The *opaque*2 (*o2*) mutation of maize markedly changes the amino acid balance, and results in a substantial increase in the lysine content [3]. Studies on rats and pigs showed that the *o2* lines are nutritionally superior to normal lines [4]. However, the *o2* mutation gives rise to a starchy, soft endosperm that is opaque when viewed on a light box. Previous studies discovered that the starchy (or opaque) phenotype is associated with reduced protein body size and lower amounts of 22-kD α-zein, a prolamin storage protein in the endosperm [5, 6]. The *o2* gene encodes a transcription factor that influences the expression of 22 kDa α-zein and many other genes [7, 8]. However, the cellular and biochemical mechanisms that cause the opaque phenotype are not well understood but recent evidence indicates that an important factor is the ratio and arrangement of zein protein isoforms within the substructure of protein bodies [9, 10].

Despite the improvement in lysine composition and nutritional value, most *o2* lines were not commercially developed, due to their low grain yields, soft, chalky endosperm, and insect susceptibility [11, 12]. To solve those problems, breeders developed modified *o2* lines by introgressing genes that alter the soft and opaque endosperm, producing a hard and vitreous endosperm while maintaining high lysine content of *o2* lines [13]. Those genes were designated *o2* modifiers (*mo2*), and the *o2* lines with those genes are called Quality Protein Maize (QPM; [14]). However, it is time-consuming to develop QPM lines, because of the difficulty of introducing multiple *mo2* loci, while simultaneously maintaining the amino acid level in kernels [13, 14].

Improvement of QPM would be easier if the key mechanisms by which the *mo2* genes produce a hard and vitreous endosperm were characterized, but so far, the location of each modifier gene and their specific downstream effects are not well understood. Genetic mapping of *mo2* revealed the linkage between a locus close to the centromere of chromosome 7 and the gene encoding 27-kD γ-zein [15]. QPMs maintain the reduced level of 22-kD α-zein as *o2* mutants, but accumulate twice to three times as much 27-kD γ-zein as wild type and unmodified *o2* lines [5, 16]. A dominant RNAi transgene was introduced into QPM lines to eliminate the expression of the gene encoding 27-kD γ-zein, resulted in an opaque phenotype in RNAi treated QPM endosperm [17]. Therefore, 27-kD γ-zein was believed to be an essential component of QPM endosperm modification associated with the vitreous phenotype. Additionally, genome-wide Quantitative Trait Locus (QTL) mapping and expression analysis identified several loci and genes associated with the vitreous phenotype of QPM, including glucose transporter, α-subunit of pyrophosphate-dependent fructose-6-phosphate 1-phosphotransferase (PFPα) and ethylene insensitive 3-like (EIL) protein [18]. And recent study showed that PFPα was coinduced with heat shock proteins (Hsps), which regulates the redox energy balance in QPM lines [19].

Apart from zeins, starch granule synthesis and structure may also influence the endosperm texture. Starch granules are composed of two types of glucan polymers: amylose, a linear alpha- 1,4 linked polymer and amylopectin, a branched glucan polymer with clusters of alpha-1,6 linkages. The organization of amylose and amylopectin forms starch granules with alternating crystalline and amorphous lamellae [20]. A mutation of gene encoding Granule-Bound Starch Synthase I (GBSS I), encoded by *waxy1* (*wx1*) prevents the production of amylose, resulting in partially opaque phenotype [21]. Also a mutation of the isoamylase-type starch debranching enzyme, encoded *sugary 1* (*su1*), results in reduced activity of both isoamylase-type and pullulanase-type debranching enzymes, and produces a vitreous and shrunken endosperm [22, 23]. Double mutants of *shrunken2* and *brittle2* decrease the activity of ADP-glucose pyrophosphorylase, causing a vitreous kernel phenotype [24]. Those mutations change the pattern of starch synthesis and starch structure, which in turn alter the kernel vitreousness.

Although both wild type and QPM have vitreous kernels, the starch structure of QPM is substantially different from its wild type counterparts [25]. Scanning electron microscopy shows that QPM starch granules form contacts between one another, which are not observed in wild type starch granules. Also, proteomic analysis of QPM lines shows an increased extractability of GBSS I from starch granules, suggesting that the interior of QPM starch granules was more accessible to solvent [25]. Those data indicate that protein-starch interactions may be different between QPM and wild type. Our goal in this study was to identify and analyze genes and their corresponding protein products associated with the vitreous endosperm phenotype of QPM lines. Previous sequence analysis of starch synthases (SS), starch branching enzymes (BE) and starch debranching enzymes (DBE) revealed that distinct alleles of four enzymes: pullulanase-type starch debranching enzyme (ZPU1); starch synthase IIa (SSIIa); starch synthase IIb (SSIIb); and starch synthase III (SSIII), are present in *mo2*. A population of recombinant inbred lines (RILs) was developed from K0326Y (QPM inbred line) crossed to W64A*o2*, followed by seven generations of self-pollination [18]. These RILs showed a broad range of phenotypes for vitreousness and allow characterization of the relationship between specific gene expression, enzyme activities, starch structure and kernel vitreousness. In this study, these RILs were used to analyze the role of pullulanase and SSIII on kernel modification. We found that the activity of pullulanase-type starch debranching enzyme was positively correlated with the kernel vitreousness and SSIII may influence pullulanase to affect starch structure and properties. Therefore, we propose that pullulanase and SSIII could play an important role in promoting formation of vitreous endosperm by altering the fine structure of starch.

## Results

### Multiple Alignment and Restriction Analysis of *Zpu1* Alleles

**Fig 1:**
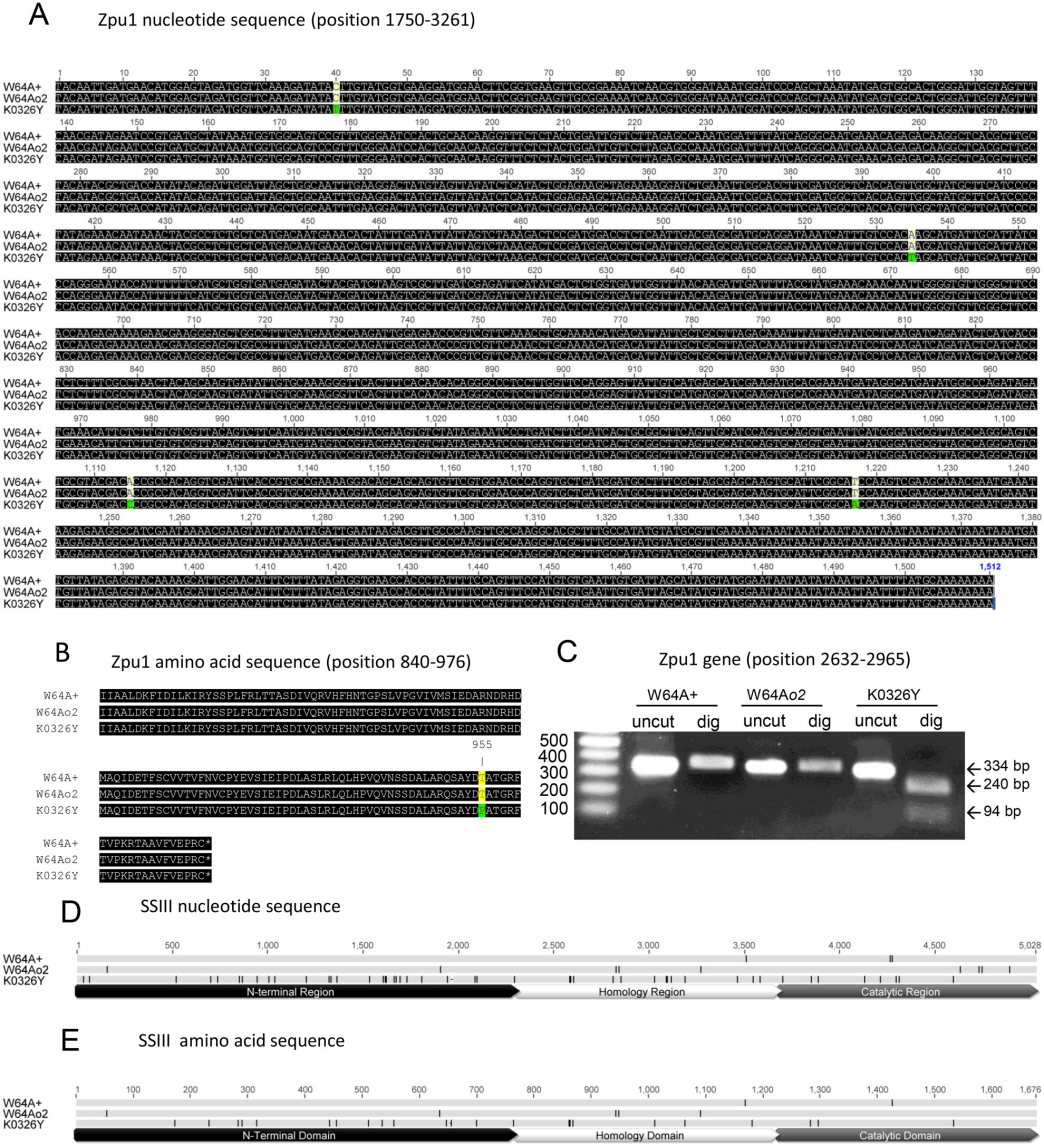
Sequence analysis of maize Zpu1 and *SSIII* genes. (A) Multiple alignments of Zpu1 gene sequences between W64A+, W64A*o2* and K0326Y. The highlighted positions showed four single nucleotide polymorphisms in K0326Y. (B) Multiple alignments of translated amino acid sequences of pullulanase. One amino acid difference (highlighted) was found in K0326Y due to the substitution of nucleotide from A to C at position 2864. (C) Restriction analysis of a 3’ end fragment (position 2632–2965) of the Zpu1 gene. The gel bands represent the size of fragments before (uncut) and after digestion (dig) with BslI. (D) Multiple alignments of *SSIII* gene sequences between W64A+, W64A*o2* and K0326Y. Hash marks on each sequence showed nucleotide polymorphisms. Prior studies identified three regions of the nucleotide sequences: N-terminal region (base 1–2304), homology region (base 2305–3679), and catalytic region (base 3680–5025) [28]. Full nucleotide sequence alignments are provided in S1 Fig. (E) Multiple alignments of translated amino acid sequences of SSIII, and hash marks on each sequence showed amino acid changes. Sequence annotation showed three domains of the amino acid sequences: N-terminal domain (amino acid 1–768) and homology domain (amino acid 769–1226), and catalytic domain (amino acid 1227–1674). Full protein sequence alignments are provided in S2 Fig.

The full length mRNA of the *Zpu1* gene has 3,261 nt (acc. AF080567,) [26]. Full length cDNA contigs for Zpu1 from W64A+, W64A*o2* and K0326Y were assembled from 8 overlapping RT-PCR fragments. Multiple sequence alignment of full-length *Zpu1* cDNA sequences from W64A+, W64A*o2* and K0326Y was performed using Geneious 5.6.4. The results showed that W64A+ (acc. AF080567) and W64A*o2* (acc. KP872821; W64A*o2*-derived *Zpu1* allele) had identical *Zpu1* alleles, while the *Zpu1* allele in K0326Y (acc. KP872822; QPM-derived *Zpu1* allele) had 4 single nucleotide polymorphisms (SNPs; Fig 1A). Three of the SNPs were silent, whereas the A→C SNP at position 2864 introduced a change in amino acid sequence from threonine to proline (Fig 1B). This SNP also created a new *Bsl*I restriction endonuclease site [27]. A DNA fragment located at the 3’ end of *Zpu1* gene (nucleotides 2632–2965) was amplified, purified and digested by *Bsl*I to confirm the presence of the SNP. The size did not change for W64A+ and W64A*o2*, while K0326Y generated two fragments 240 bp and 94 bp, respectively, indicating the presence of the QPM SNP (Fig 1C).

### Multiple Alignment of *SSIII* Alleles

The full length coding region of the *SSIII* gene (5025 bp) from W64A*o2* (5028 bp) and K0326Y were cloned into the pRSET-C vector and sequenced. Their sequences were aligned with the full length W64A+ *SSIII* cDNA sequence (acc. JF273457) [28]. W64A+ and W64A*o2* (acc. KR350619; W64A*o2*-derived *SSIII* allele) had similar *SSIII* gene sequences, except for the presence of 12 SNPs 5 of which were silent. In contrast, the K0326Y *SSIII* gene (acc KR350620; QPM-derived *SSIII* allele) showed much more variation throughout the sequence (Fig 1D; S1 Fig) with 51 SNPs and a single 3 bp deletion. The alteration of some nucleotides resulted in change in the resulting amino acid sequence. Compared with the conceptual translation of the W64A-derived *SSIII* sequence, the QPM-derived SSIII sequence had 25 amino acid substitutions and one amino acid deletion at position 654 in the N-terminal domain (Fig 1E; S2 Fig).

### Pullulanase Activity and Starch Synthase III Abundance

#### Parental Lines

**Fig 2:**
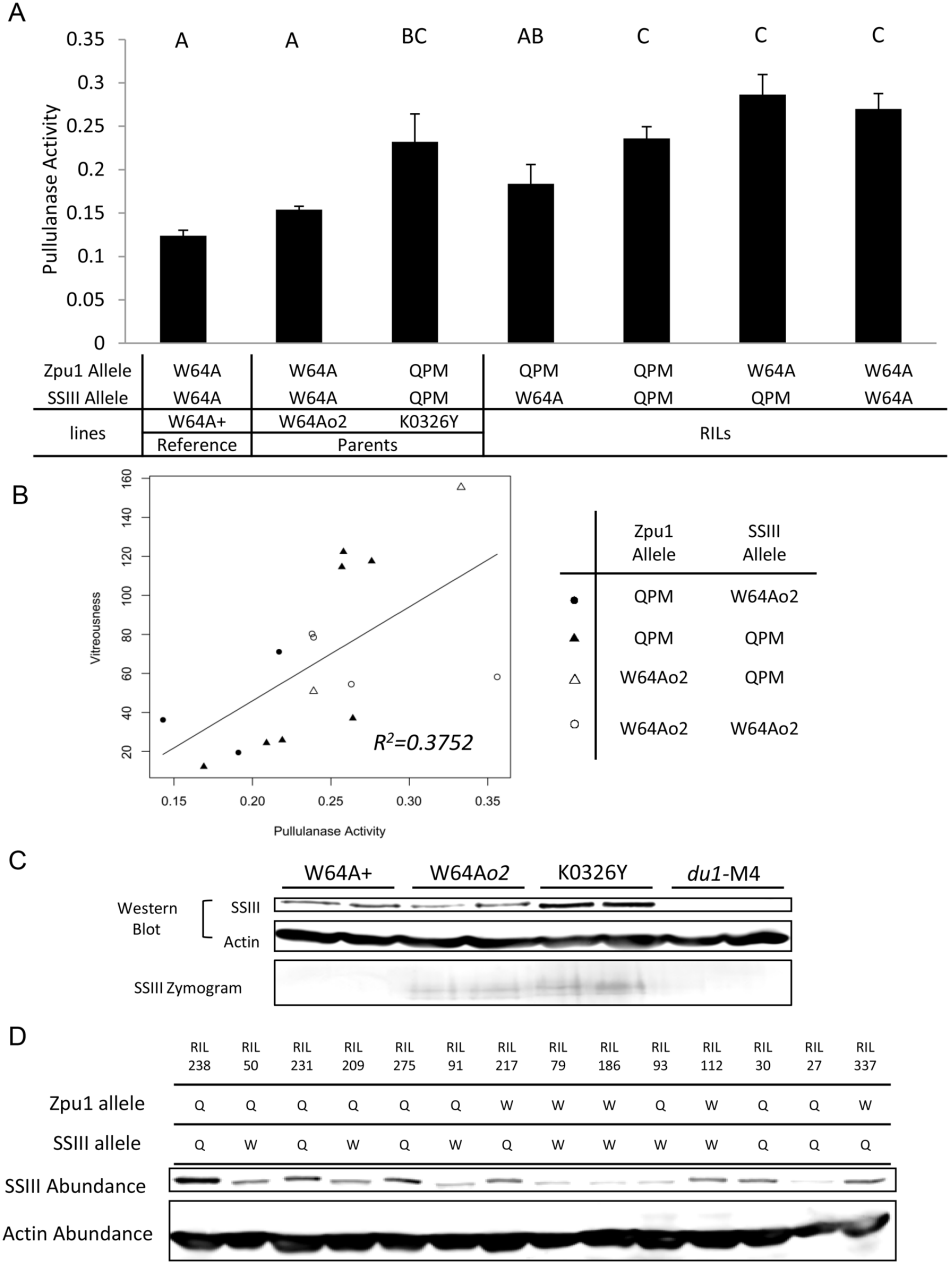
Pullulanase activity and SSIII abundance in parental lines and RILs. (A) Pullulanase activity of W64A+, W64A*o2*,K0326Y and RILs with four Zpu1-SSIII genotypes. Pullulanase activity was measured by the spectrophotometer at 490 nm absorbance (y-axis). For the first three columns, each represents mean pullulanase activity of three independent ears of the corresponding lines; and for the remaining four columns, each represents mean pullulanase activity of all RILs with the corresponding genotype from three independent ears of each individual RIL. The letters above each column represent statistically significant differences among the lines with *p*<0.05 by pairwise two-tailed t-test. Columns with the same letter are not significantly different from one another. The error bars represent standard error. (B) Positive correlation between pullulanase activity and kernel vitreousness. The significance level of the correlation was tested by ANOVA of the slope at *p*<0.05. Each data point represents mean pullulanase activity of three independent ears of each individual RIL. (C) SSIII abundance and SSIII activity of W64A+, W64A*o2*, K0326Y and *du*1M4. SSIII abundance was tested by SDS-PAGE followed by Western blot (Images of the whole blots and SDS-PAGE gels are provided in S4 and S6 Figs). SSIII activity was tested by native PAGE followed by a zymogram for the RILs. (Image of whole zymogram gel is provided in S4 Fig) (D) Western blot of SSIII among RILs homologous for W64Ao2 (W) or QPM (Q)–derived *Zpu1* or *SSIII* alleles. The order of samples corresponded to their kernel vitreousness, from most opaque to most vitreous (left to right; images of whole blots and SDS-PAGE gels are provided in S5 and S7 Figs).

The activity of pullulanase and the abundance of SSIII were tested by quantitative enzyme activity assays and Western blots, respectively. In the parental lines of the RIL population, pullulanase enzyme activity of K0326Y was significantly higher than W64A*o2*, and W64A+ (Fig 2A). The abundance of SSIII in K0326Y was significantly higher than W64A*o2* and W64A+, but W64A*o2* and W64A+ did not show any significant difference in SSIII protein abundance; whereas du1-M4, an *SSIII*-null mutant, had no detectable SSIII (Fig 2C). SSIII enzyme activity in the parental lines was tested by a zymogram based on native PAGE, and showed that K0326Y had higher SSIII activity than W64A*o2*. W64A+ and W64A du1-M4 did not have any detectable SSIII activity (Fig 2C).

#### Recombinant Inbred Lines

Pullulanase activity and SSIII abundance were also tested in RIL populations homozygous for either the W64A*o2* allele or the QPM allele of the *Zpu1* gene and *SSIII* gene, respectively. The pullulanase activity was influenced by both *Zpu1* gene allele itself and also by the *SSIII* gene allele. RILs homozygous for QPM-derived alleles of *Zpu1* gene and W64A*o2*-derived alleles of *SSIII* gene (Q-W) showed significantly lower pullulanase activity than others. Also, among the RILs with the same *Zpu1* allele, those homozygous for QPM-derived alleles of *SSIII* gene tended to have higher pullulanase activity than those homozygous for W64A*o2*-derived alleles of the *SSIII* gene (Fig 2A). Pullulanase activity showed an approximately threefold variation, and the kernel vitreousness of the RIL population had an approximately 50-fold variation [18]. The activity of pullulanase was positively correlated with kernel vitreousness (Fig 2B), based on ANOVA analysis and correlation tests (*R* ^*2*^ = *0*.*3762*, *p*<*0*.*05*), indicating that pullulanase activity could be a candidate factor that influences the vitreous phenotype. Previous studies showed that kernel vitreousness, density and hardness were correlated with one another [18]. However, our data showed that the pullulanase activity was not significantly correlated with kernel density or hardness (S3 Fig), suggesting that other factors affect hardness and density. SSIII abundance was highly dependent on which *SSIII* allele was present in the genetic background. The abundance of SSIII was higher in RILs homozygous for the QPM allele compared to those homozygous for W64A*o2* alleles (Fig 2D). However, the Zpu1 allele in the background did not appear to have a significant effect on SSIII abundance. The order of RILs in the Fig 2D corresponded to their kernel vitreousness, from most opaque to most vitreous. However, the SSIII abundance did not show significant correlation with kernel vitreousness, suggesting that SSIII was not a factor directly affecting the kernel vitreousness.

### Differential Scanning Calorimetry

Differential scanning calorimetry (DSC) was used to evaluate the starch gelatinization and measure the starch thermal properties [29, 30]. When heated in water, the starch gelatinization can be reflected as a curve of endotherm. The temperature at which starch begins melting is called the onset temperature, and the temperature at which starch reaches to an order-disorder transition point with the peak endotherm is called the maximum temperature. The area of the curve is proportional to the enthalpy and is positively related to the degree of crystallinity [29, 31].

**Fig 3:**
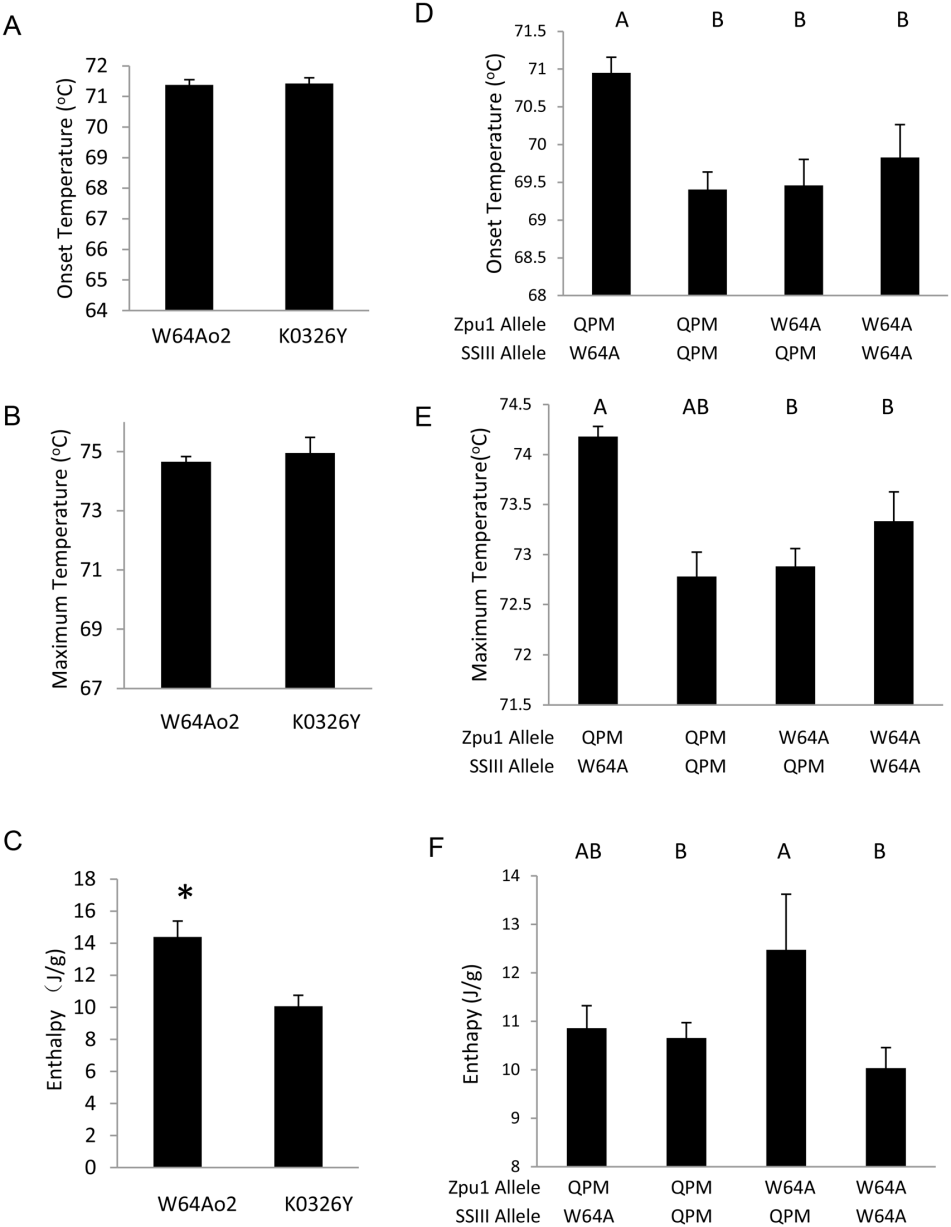
Thermal properties of starch. (A-C) Comparison of starch thermal properties, including onset temperature, maximum temperature and enthalpy, between W64A*o2* and K0326Y. Significant level indicated by asterisks at p<0.05 by two-tailed *t* test (D-F) Comparison of the starch thermal properties among RILs. For parental lines, each column represents mean thermal property values of three independent ears of corresponding lines; and for RILs, each column represents mean thermal property values of all RILs of the corresponding genotype with three independent ears for each individual RIL. The letters above each column represent statistically significant differences among the lines for *p*<0.05 by pairwise two-tailed t-test. Columns sharing the same letter are not significantly different from one another. The error bars represent standard error.

DSC analysis of the parental lines W64A*o2* and K0326Y showed that there was no significant difference between *o2* and QPM parental lines for onset and maximum endotherm, but K0326Y had significantly lower enthalpy (Fig 3A–3C), indicating that QPM starch granules have lower overall crystallinity. In addition, DSC analysis of RILs with varying vitreousness showed that the thermal properties of the starch were influenced by both pullulanase and SSIII (Fig 3D–3F). The onset temperature and maximum temperature of the RILs homozygous for QPM-derived *Zpu1* alleles and W64A*o2*-derived *SSIII* alleles (Q-W) were significantly higher than others (Fig 3D and 3E). Also, among the RILs with homozygous QPM-derived *Zpu1* alleles, those homozygous for QPM-derived *SSIII* alleles (Q-Q) showed significantly lower onset and maximum temperature than those homozygous for W64A*o2*-derived *SSIII* alleles (Q-W). Among the RILs homozygous for the W64A*o2*-derived *Zpu1* alleles, those with homozygous QPM-derived *SSIII* alleles (W-Q) showed significantly higher enthalpy than those homozygous for the W64A*o2*-derived *SSIII* alleles (W-W). Likewise, among the RILs homozygous for the QPM-derived *SSIII* alleles, those homozygous for the QPM-derived *Zpu1* alleles (Q-Q) had significantly lower enthalpy than those with W64A*o2*-derived *Zpu1* alleles (W-Q). These data suggest that pullulanase and SSIII could influence the activity of each other resulting in different glucan chain length and crystallinity of starch granules, which in turn influence the starch thermal properties and starch granule structure.

### Glucan Chain Length Distribution

**Fig 4:**
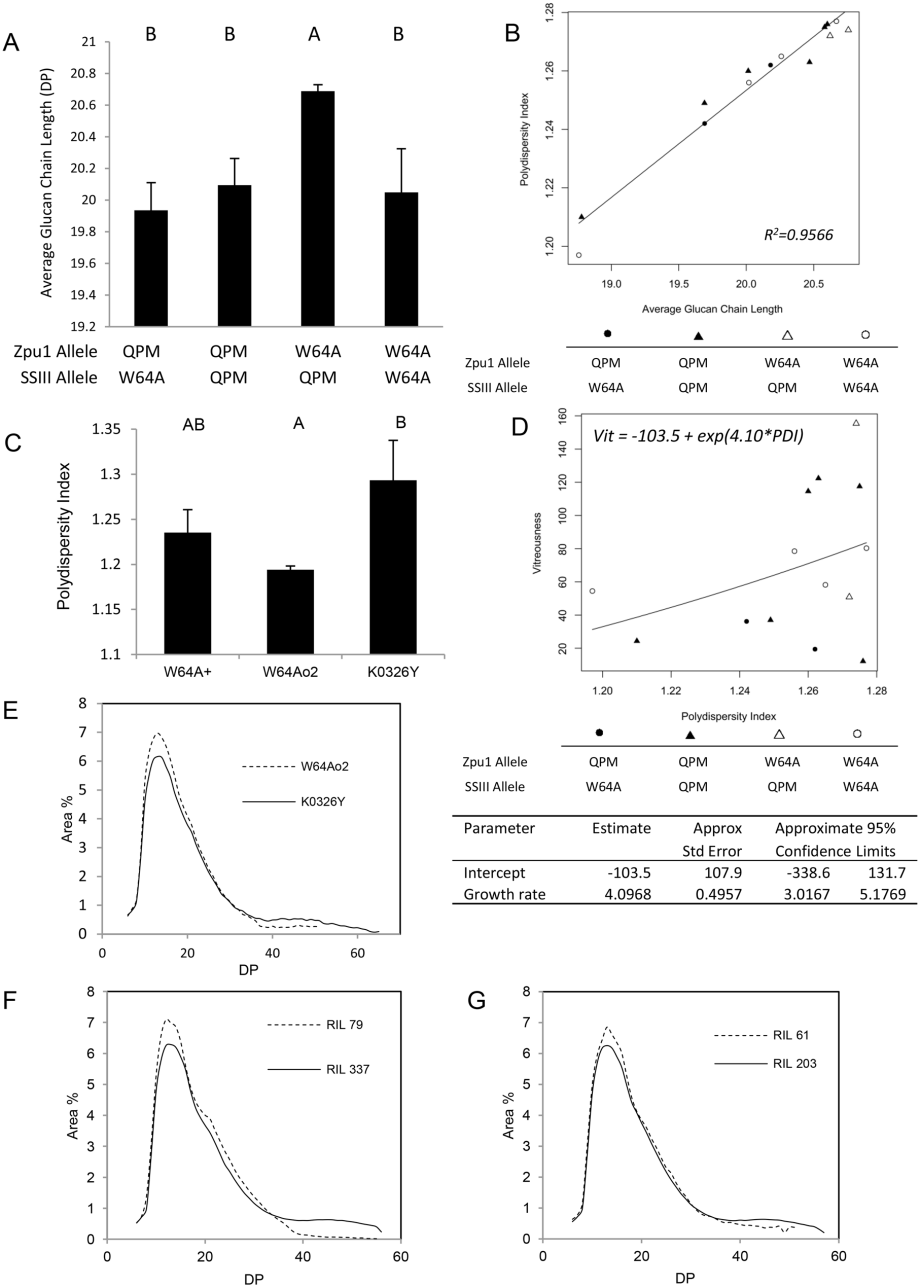
Average glucan chain length and polydispersity index (PDI). (A) Comparison of the average glucan chain length among RILs. Each column contains multiple lines homozygous for the parental alleles (W64A*o2* or QPM) of *Zpu1* or *SSIII*. Each column represents mean glucan chain length of all RILs of the corresponding genotype with three independent ears of each individual RIL. (B) Positive correlation between average glucan chain length and PDI. The correlations are significant, tested by ANOVA of the slope at *p*<0.05. Each data point represents mean glucan chain length of three independent ears of each individual RIL. (C) Comparison of PDI between parental lines (W64A+, W64A*o2* and K0326Y). Each column represents the mean PDI value of three independent ears of corresponding lines. The letters above each column of (A) and (C) represent statistically significant differences among the lines for *p*<0.05 by each pair t test. Columns sharing the same letter are not significantly different from one another. The error bars represent corresponding standard error. (D) Nonlinear regression analysis between PDI and kernel vitreousness among RILs, test statistics are listed below. The relationship was significantly positive, because 0 was excluded from the 95% confidence interval of Growth Rate, indicating that the Growth Rate was greater than 0 at *p*-value = 0.05 level. Each data point represents mean PDI of three independent ears of each individual RIL. (E-G) Glucan chain length distribution between opaque lines (dash curves) and vitreous lines (solid curves).

In order to understand what factors more directly influence the kernel vitreousness, the glucan chain length distribution of debranched amylopectin from the RIL populations was measured via fluorescence-assisted capillary electrophoresis (FACE). The results showed that the average degree of polymerization (DP; the number of α-D-glucopyranosyl units in glucan chains) was affected by both pullulanase and SSIII. The RILs homozygous for both W64A*o2*-derived *Zpu1* alleles and QPM-derived *SSIII* alleles (W-Q) showed the highest average glucan chain length among all selected RIL samples. Also, among the RILs homozygous for W64A*o2*-derived *Zpu1* alleles, those homozygous for W64A*o2*-derived *SSIII* alleles (W-W) showed significant lower average glucan chain length than those homozygous for QPM-derived *SSIII* alleles (W-Q). Among the RILs homozygous for QPM-derived *SSIII* alleles, those homozygous for W64A*o2*-derived *Zpu1* alleles (W-Q) showed significantly higher average glucan chain length than those homozygous for QPM-derived *Zpu1* alleles (Q-Q or Q-W) (Fig 4A). The average glucan chain length among those four types of RILs had similar trend to their enthalpy values (Fig 3F), indicating the glucan chain length could influence the crystallinity of starch granule, which in turn affects the enthalpy change during gelatinization.

DP and corresponding percent peak area were used to calculate the polydispersity index (PDI). The PDI measures the width of molecular weight distributions [32], and the PDI of glucan chain length was highly correlated with average glucan chain length (Fig 4B), making PDI a good parameter to describe the distribution of glucan chain length. The PDI analysis of parental lines showed that the PDI of K0326Y was significantly higher than that of W64A*o2* (Fig 4C). The trend correlated well with the comparison of kernel vitreousness between W64A+, W64A*o2* and K0326Y [25]. Also, the PDI of RILs showed a significant positive non-linear exponential relationship with kernel vitreousness (Fig 4D), based on non-linear regression modeling using SAS 9.2 (SAS Institute Inc., Cary, NC). According to the non-linear analysis, the approximate 95% confidence interval of Growth Rate (lower 3.0167, upper 5.1769) did not include 0, indicating that the Growth Rate was greater than 0 at *p*-value = 0.05 level. Fig 4E, 4F and 4G showed the comparison of glucan chain length distribution between opaque (low vitreousness) lines and vitreous (high vitreousness) lines among parental lines and some RILs. From the figures, the curves of vitreous lines (K0326Y, RIL 337 and RIL203) were flatter, with longer tail and lower peak, than corresponding opaque lines (W64A*o2*, RIL 79 and RIL 61), indicating vitreous lines had higher glucan chain heterogeneity (PDI) than opaque lines, which was consistent with the relationship between vitreousness and PDI on Fig 4D.

### Scanning Electron Microscopy

**Fig 5:**
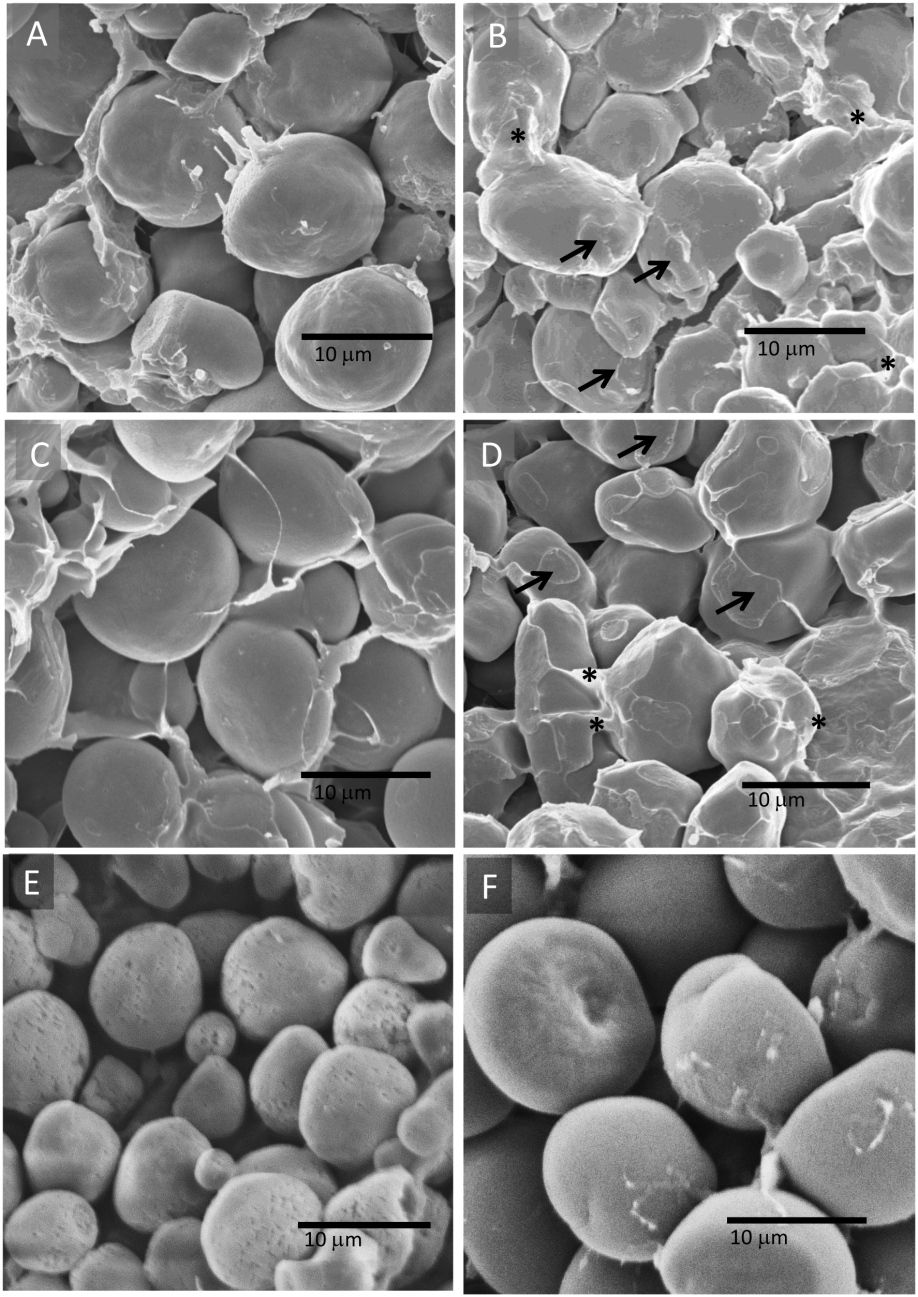
Scanning electron microscopy of starch granules from parental lines and RILs. (A) Smooth starch granules in W64A*o2* endosperm with a little matrix surrounding them. (B) Contacts (arrows) and interconnections (asterisks) formed between adjacent starch granules in K0326Y endosperm. (C) Starch granules in the endosperm of the opaque RIL 217 showed similar smooth surface as in W64A*o2*. (D) Starch granules in the endosperm of RIL 27 showed similar contact and interconnection structure as in K0326Y. (E-F) Starch granules in the endosperm of RIL 209 (E) and RIL 112 (F) showed similar smooth surface as in W64A*o2*.

Scanning electron microscopy was performed to analyze the gross structure and organization of starch granules in W64A*o2*, K0326Y, RIL 217, RIL 27, RIL 209 and RIL 112 endosperms. The starch granules of W64A*o2* were smooth and separate, except for a small amount of matrix material surrounding them and had fairly uniform large size (Fig 5A), whereas there were many contacts (arrows) and interconnections (asterisks) forming between adjacent starch granules in K0326Y endosperm and the granules varied in size (Fig 5B). RIL 217 homozygous for the W64A*o2*-derived *Zpu1* alleles and homozygous for the QPM-derived *SSIII* alleles (W-Q) had opaque kernels, and had fewer and less extensive contacts between adjacent starch granules than K0326Y, but more material was present between the starch granules than in W64A*o2* or some of the other RILs (Fig 5C). In contrast, RIL 27 homozygous for the QPM-derived *Zpu1* alleles and homozygous for the QPM-derived *SSIII* alleles (Q-Q) had vitreous kernels, and showed marked contacts and interconnections between starch granules (Fig 5D), similar to K0326Y. In addition, RIL 209 homozygous for the QPM-derived *Zpu1* alleles and homozygous for the W64A*o2*-derived *SSIII* alleles (Q-W) had opaque kernels, and showed smooth and separate starch granules like W64Ao2 but the starch granules varied in size like K0326Y (Fig 5E).Whereas RIL 112 homozygous for the W64A*o2*-derived *Zpu1* alleles and homozygous for the W64A*o2*-derived *SSIII* alleles (W-W) had semi-vitreous kernels, but showed smooth and separate starch granules (Fig 5F) similar to opaque kernels.

## Discussion

The modifier genes in QPM alter the soft and starchy endosperm in *o2*, giving rise to a hard and vitreous phenotype [13]. The study of modifier genes and their specific protein products is key to understand the mechanism responsible for the modification of the *o2* endosperms. Previous sequence analysis of starch biosynthesis genes showed that *SSIIa*, *SSIIb*, *SSIII* and *Zpu1* were hypothesized to be the candidate genes that influence the kernel phenotypes of QPM lines. Some evidence showed that the QPM amylopectin branching pattern was most similar to the *SSII* mutant in maize and rice [25], and the *SSIIa* mutation (*sugary2*, or *su2*) increased the proportion of short amylopectin branches [25, 33]. Although those starch synthases might have potential effect on kernel phenotypes, this study focused on the influence of pullulanase and SSIII on kernel vitreousness, because they were the only two enzymes that appeared to be associated with changes in vitreousness.

Pullulanase-type debranching enzymes hydrolyze the α-1,6 glycosidic bonds of pullulan, a linear polymer of maltotriose units produced during starch metabolism [26]. In maize, pullulanase activity is mainly observed in developing kernels. A null mutation of pullulanase, *Zpu1-204*, alters the normal starch catabolism and accumulates branched maltooligosaccharides, although compared with the wild type, it does not exhibit any obvious morphological differences in kernels [34]. A *su1/Zpu1-204* double mutation, however, produces kernels with wrinkling, vitreous crown, even extending further into the central region [34], suggesting that both pullulanase and isoamylase are involved in starch branching pattern editing and determination, and can affect the endosperm texture and kernel phenotype.

SSIII is an important starch synthase involved in glucan chain elongation and also functions as a scaffolding protein that brings together multiple components of the starch biosynthetic pathway, and evidence showed that SSIII mutants altered the branching pattern and resulted in larger amylopectin clusters [35, 36, 37]. The protein binding activity was found to me mediated at the highly diverse N-terminal domain and the relatively conserved homology domain, and glucan chain length elongation was mediated by the highly conserved catalytic domain at the C-terminal region [28, 36, 38]. The sequence analysis revealed some amino acid changes in the N-terminal domain and in the homology domain of W64A*o2*. It is not clear if the polymorphisms between W64a+ and W64A*o2* are the result of new mutations arising in the many generations that the lines have been used by independent laboratories or if the W64A*o2* allele is a remnant from a parental line used for introgression of the *o2* mutation into the W64A background. In contrast, the QPM-derived SSIII had amino acids altered in the catalytic domain compared with the W64A-derived SSIII (Fig 1E), which could be one of other factors causing the increase of SSIII catalytic activity in K0326Y, apart from higher enzyme abundance (Fig 2C). The amino acid changes in the N-terminal domain and homology domain of QPM-SSIII may influence the formation of starch biosynthetic complex by changing the binding activity to other starch biosynthetic enzymes (Fig 1E). The underlying mechanisms need to be further characterized, but SSIII could be one factor that influences the endosperm texture and kernel phenotype.

This study showed that there was significant positive correlation between pullulanase activity and kernel vitreousness according the quantitative pullulanase activity assay, as well as kernel vitreousness assays of RILs generated by crossing K0326Y QPM lines to W64A*o2* opaque lines (Fig 2B). The parental lines of RILs, K0326Y and W64A*o2*, showed significantly different pullulanase activity. With higher kernel vitreousness, K0326Y had higher pullulanase activity than W64A*o2* with lower kernel vitreousness (Fig 2A). Multiple sequence alignment revealed that the QPM-derived *Zpu1* allele was different from the W64A*o2*-derived allele in some SNPs, one of which altered the threonine to proline (Fig 1A and 1B). Compared with threonine, proline has a unique closed ring structure making it difficult to form many of the main-chain conformations and adopt normal helical conformations [39], thus it is possible that the substitution might alter the pullulanase 3D structure, which in turn affects pullulanase activity.

Pullulanase activities of parental lines and RILs were compared in Fig 2A. The pullulanase activity of K0326Y was consistent with RILs homozygous for QPM-derived Zpu1 allele and *SSIII* allele (Q-Q), but W64A*o2* was not consistent with RILs homozygous for W64A*o2*-derived Zpu1 allele and *SSIII* allele (W-W). Also from Fig 2B, pullulanase activity showed a quantitative trait pattern, but its allele was not mapped as one of the QTLs reported by Holding et al. [18], suggesting that there might be one or more upstream QTL factors affecting pullulanase activity. These factor(s) might have segregated during the formation of RILs, which caused the discrepancy between the pullulanase activities of W64A*o2* and W-W RILs.

In addition, SSIII also could affect the pullulanase activity. The RILs homozygous for QPM-derived *SSIII* alleles had higher SSIII abundance than those homozygous for W64A*o2*-derived *SSIII* alleles (Fig 2C and 2D), and among those RILs with same *Zpu1* genotype, those homozygous for QPM-derived *SSIII* alleles tended to have higher pullulanase activity than those homozygous for W64A*o2*-derived *SSIII* alleles (Fig 2A). Specifically, RILs showed in both 3rd and 4th column had QPM derived pullulanase allele (Q), but those with QPM-derived *SSIII* allele (Q) have significant higher pullulanase activity than those with the W64A*o2*-derived *SSIII* allele (W); whereas RILs showed in last two columns had W64A*o2*-derived pullulanase allele (W), although they didn’t have significant difference, those with QPM derived *SSIII* allele (Q) still had a relative trend of higher pullulanase activity than those with W64A*o2-*derived *SSIII* alleles (W) suggesting that the high abundance of SSIII might promote the activity of pullulanase. However, there was no significant correlation between kernel vitreousness and SSIII abundance (Fig 3D), indicating that SSIII might not influence the kernel vitreousness directly. The structure of amylopectin in these lines were analyzed by DSC, glucan chain length measured by FACE and the PDI was calculated based on the glucan chain length distribution. The DSC analysis of RILs showed that pullulanase and SSIII were two of the factors affecting the starch thermal properties. Data showed that RILs homozygous for the same type of *Zpu1* and *SSIII* alleles (both derived from either W64A*o2* or QPM, Q-Q or W-W) did not have significantly different onset and maximum temperature (Fig 3D and 3E), which was consistent with the corresponding parental data (Fig 3A and 3B), whereas those homozygous for different types of *Zpu1* and *SSIII* allele (Q-W or W-Q) showed significant differences (Fig 3D and 3E), which could be explained by a counterbalance effect between pullulanase and SSIII. Previous studies revealed that the *Zpu1* mutant reduced the amount of water-soluble polysaccharides (WSP) [33], suggesting that higher pullulanase activity could increase the amount of WSP. Pullulanase functions to hydrolyze α-1, 6 linkages in small, branched polysaccharides, producing short water-soluble glucans consist mainly of maltose, maltotriose, and maltotetraose [33]. These small glucans could be recycled and used as building blocks for starch chain elongation and branch formation by collaborating with SSs and SBEs. Therefore, higher pullulanase activity tends to have higher capacity to produce small glucans used to form short branches. SSIII, however, functions to elongate glucan chains, so higher the amount of SSIII tends to have higher capacity to produce long branches, which therefore might counterbalance the effect of pullulanase to some extent. In addition, the onset and maximum temperature did not show a strong correlation with average glucan chain length (Fig 4A), indicating that these parameters are not only affected by glucan chain length, as many studies have shown [39, 40, 41], but also affected by other factors, such as branching pattern, polydispersity and starch composition [42].

The enthalpy of starch gelatinization reflects the starch crystallinity. This study showed that average glucan chain length had similar trend comparing with enthalpy among four types of RILs (Figs 3F and 4A), suggesting that glucan chain length could be one of the important factors associated with the starch crystallinity, which was consistent with prior studies [43]. Average glucan chain length was also dependent on both pullulanase and *SSIII* alleles (Fig 4A), and had strong correlation with PDI (Fig 4B), which reflects the heterogeneity of glucan chains. Prior studies revealed that kernels of W64A*o2* had significantly lower vitreousness than W64A+ and K0326Y [25], and this trend correlated well with the PDI among parental lines (Fig 4C). Similar results were observed in analysis of the RILs (Fig 4D), which suggests that the heterogeneity of glucan chains could be an important factor contributing to the kernel vitreousness.

Scanning electron microscopy analysis revealed dramatic differences in starch granules between opaque and vitreous kernels. In the study, compared with W64A*o2*, RIL217 (W-Q), RIL 209 (Q-W) and RIL 112 (W-W) with smooth and separate starch granule (Fig 5A, 5C, 5E and 5F), both K0326Y and RIL 27 (Q-Q) had contacts and interconnections between adjacent starch granules (Fig 5B and 5D). These observations are consistent with previous findings using independently developed modified lines CM105*o2*, CM105*mo2*, RIL 5 (opaque kernels) and RIL 32 (vitreous kernels) [25]. Together these data suggest that the contacts and interconnections between starch granules were associated with the formation of vitreous endosperms in QPM lines and RILs with QPM-derived *Zpu1* and *SSIII* alleles, although the formation and composition of those contacts and interconnections have not yet been fully characterized.

In conclusion, the study revealed that pullulanase activity was positively correlated with the kernel vitreousness, and SSIII could influence kernel vitreousness indirectly by affecting the pullulanase activity and by altering the starch fine structure. The data showed that pullulanase and SSIII influenced glucan chain length distribution, resulting in diverse PDI, thermal properties and starch granule surface profiles. Therefore it is possible that pullulanase and SSIII represented two factors associated with the formation of vitreous kernel phenotype. Nevertheless, more studies are needed to elucidate the mechanism of any pullulanase-SSIII interaction and the detailed genetic mechanism for pullulanase and SSIII to promote kernel vitreousness.

## Materials and Methods

### Genetic Materials

All maize lines in the study were grown and harvested in summer, 2011 in Elm Mott, TX. The parental lines used in the pullulanase activity assay, SSIII abundance assay, DSC and the measurement of glucan chain length distribution were W64A*o2* (an *opaque 2* mutant line in the W64A inbred, an early maturing dent developed by the Wisconsin Agricultural Experiment Station) and K0326Y (a tropical QPM inbred line developed in South Africa by Hans Geevers) and harvested at 18 days after pollination (DAP). 14 recombinant inbred lines (RILs) were originally obtained from University of Arizona. RILs were derived from F2 kernels of a K0326Y x W64A*o2* cross, followed by seven generations of self-pollination, which manifest a broad range of soft, opaque to vitreous, hard phenotypes [18]. RILs involved in the assay mentioned above were also harvested at 18 DAP. Additionally, W64A+ and the SSIII null mutant W64A*du1*-M4 were harvested at 18 DAP and used as references for assays on parental lines. All developing kernels were frozen in liquid nitrogen and stored at -80°C.

### Full Length Zpu1 cDNA Sequencing and Sequence Alignment

For each reverse transcription reaction, isolated *Zpu1* mRNA (1μg) was mixed with 0.5 μg of Oligo(dT)15 primer and RNase-free H_2_O to make final volume of 5μL. The mRNA/primer mixture was preheated at 70°C for 5 min and chilled on ice for 5 min, and then mixed with reverse transcription mixture containing 4 μL of ImProm-II (Promega Inc. Madison, WI) 5X Reaction Buffer, 1 μL of dNTP, 1.5 μL of 50 mM MgCl2, 0.5 μL of Recombinant RNasin Ribonuclease Inhibitor, 1 μL of ImProm-II Reverse Transcriptase (Promega Inc.) and 7 μL RNase-Free H_2_O (to a final volume of 15 μL). Reverse transcription was performed in an S1000 Thermal Cycler (Bio-Rad Laboratories, Inc.) with priming at 25°C for 5 min, reverse transcription at 42°C for 60 min and inactivation at 70°C for 15 min.

Eight pairs of primers were designed via primer designing tool using Geneious (v. 5.6.4, Biomatters, Aukland, NZ) and synthesized by the Midland Certified Reagent Company (Midland, TX). The sequences are listed in S1 Table. The PCR reaction mixture contained 10 μL of 5X Phusion HF Buffer (New England BioLabs, Inc. Ipswich, MA), 1 μL of dNTP, 1 μL of forward and reverse primers, 0.5 μL of Phusion High-Fidelity DNA Polymerase (New England BioLabs, Inc.), 2 μL of cDNA template, 1.5 μL of DMSO and diH_2_O to make final volume of 50 μL. PCR was also performed in an S1000 Thermal Cycler for 40 cycles with pre-denaturation at 98°C for 1 min, denaturation at 98°C for 20s, annealing at 60°C for 25s, extension at 72°C for 45s, and final extension at 72°C for 10 min. The PCR products were tested by 1% agarose gel electrophoresis at 120V for 30 min, and purified via Illustra GFX PCR DNA and Gel Band Purification Kit (GE healthcare) following the manufacturer’s manual. The purified fragments were sequenced by Macrogen Inc. (Rockville, MD). Geneious was used to assemble sequence of fragments, to align them with B73 reference sequence from MaizeGDB (http://www.maizegdb.org/) and to perform the multiple alignment between W64A+, W64A*o2* and K0326Y with the default parameter setup.

### Restriction Analysis of Zpu1 Alleles

For each restriction reaction, 1 μg of *Zpu1* gene 3’ end fragment, the PCR product by the primer *Zpu1*_3'F and *Zpu1*_3'R (S1 Table), was mixed with 0.5 μL *Bsl*I restriction enzyme, 5 μL CutSmart Buffer (New England BioLabs, Inc.) and diH2O to make the final volume of 50 μL. The reaction mixture was incubated at 55°C for 1 hour. The DNA fragments before and after the treatment of *Bsl*I were loaded on 1% agarose gel and the electrophoresis was performed at 120 V for 30 min.

### SSIII Coding Region Sequencing and Multiple Alignments

Total RNA was extracted and isolated from frozen developing endosperm (18 DAP) of W64A*o2* and K0326Y following the conventional procedure according to Jia et al. [44]. The cDNA was synthesized using ImProm-I Reverse Transcription System (Promega Corp. Madison, WI) according to the manufacturer’s instructions. SSIII full-length ORF was synthesized in 25 μL reaction system with 5 μL of Phusion HF Buffer, 0.5 μL of dNTP, 0.5 μL of each primer (Du1_Forward with XhoI restriction site: ACTAACCTCGAGACCCTTCTTTTCTTCCCCTTC and Du1_Reverse with EcoRI restriction site: GCACGTGAATTCTTACAATTTGGACG-CTGAAC), 0.25 μL of Phusion High-Fidelity DNA Polymerase (NEB, Ipswich, MA), 0.75 μL of 100% DMSO, 2 μL of cDNA and up to 25 μL diH2O. The reaction condition was set as pre-denaturation 98°C 30s, denaturation 98°C 10s, annealing 60°C 25s, extension 72°C 6min, final extension 72°C 10min, and the cycle number was set as 35. The PCR product was gel purified using Illustra GFX PCR DNA and Gel Band Purification Kit (GE Healthcare, Little Chalfont, United Kingdom), and digested by XhoI and EcoRI (NEB, Ipswich, MA), then ligated with vector pRSET-C (Life Technologies, Grand Island, NY) digested by same set of restriction enzyme. The recombinant plasmid was transformed into 10-beta Competent E. coli (NEB, Ipswich, MA). Then recombinant plasmids purified from overnight culture of positive clones using EZNA Plasmid DNA Mini Kit (Omega Bio-Tek, Inc. Norcross, GA) were sent to Macrogen Inc. (Rockville, MD) for sequencing. The primers sequences for sequencing were taken from from Lin et al. 2012 [28]. The multiple alignments of nucleotide sequences and conceptual translations were performed using Geneious (v. 5.6.4, Biomatters, Aukland, NZ) and compared with the W64A+ SSIII sequence available at GenBank (acc. JF273457) [28]. The gene sequences of the W64Ao2 and K0326Y SSIII coding regions were submitted to genbank with the accession numbers KR350619 and KR350620, respectively.

### Protein Extraction and Pullulanase Activity Assay

Pericarps and embryos of developing kernels (18 DAP) from parental lines (three independent ears) and RILs (three independent ears) were removed. Their endosperms were ground into fine powder in liquid nitrogen. The powders were weighed and suspended with kernel protein extraction buffer (50 mM Tris-Acetate solution, pH 8.0, 10 mM EDTA, and 5 mM DTT, 2 μL/mg ground powder). The crude homogenates were centrifuged at 16,000 g for 10 min at 4°C, and the supernatants with water-soluble proteins were collected. The concentration of total water-soluble proteins was determined by NanoDrop Spectrophotmeter ND-100 (Thermo Fisher Scientific, Inc.) at 280 nm and assuming an OD of 1.0 equaled 1 mg/ml total protein in the extracts.

The kernel protein extracts were diluted with sodium acetate buffer (200 mM sodium acetate, pH 5.0 adjusted by glacial acetic acid) to make the final concentration of total water-soluble proteins 10 mg/mL and the final volume of the extract solution 50 μL. Red-pullulan (Megazyme International. Wicklow, Ireland) was used to measure the activity of pullulanase. A 2% [w/v] red-pullulan, 50 mM KCl solution and diluted kernel protein extracts were pre-equilibrated at 40°C for 5 min. Then 50 μL extract was mixed with 50 μL of red pullulan solution. The mixture was incubated at 40°C for 10 min for pullulanase digestion. The reaction was terminated with 125 μL of 100% ethanol to precipitate undigested red-pullulan molecules for 10 min. The precipitate was spun down at 16,000 g for 10 min at room temperature. The supernatant, containing the ethanol-soluble small dyed oligosaccharides, was collected and 80 μL of the supernatant was transferred into a 96-well plate, and absorption measured at a wavelength of 490 nm.

### Western Immunoblotting and Zymogram Analysis of SSIII

Kernel protein extracts were diluted with distilled water to total protein concentration of 15 mg/mL before being loaded onto a 7.5% denaturing gel with 0.4% [w/v] SDS. SDS-PAGE was performed at room temperature for 2.5 hours at 120V. Then the gel was transferred onto nitrocellulose sheets using standard methods [45]. The antisera used in western immunoblotting were rabbit anti-SSIII at 1:2,000 dilution and mouse anti-actin (Sigma-Aldrich Co. St. Louis, MO) with 1:4,000 dilution as control. The secondary anti-sera were HRP-Goat anti-Rabbit (Life Technologies, Grand Island, NY) with 1:30,000 dilution and HRP-Goat anti-Mouse with 1:40,000 dilution (Life Technologies, Grand Island, NY). Bands were visualized using a chemiluminescence substrate on an ImageQuant LAS 4000 (GE Healthcare, Piscataway, NJ). For zymogram analysis, kernel protein extracts were diluted with distilled water to a total protein concentration of 12mg/mL before being loaded onto a 7.5% native polyacrylamide gel with 0.5% oyster glycogen. Native PAGE was performed at 4°C for 4 hours at 120V. The gel then was incubated in starch synthase reaction buffer (100 mM Bicine, pH 8.0, 0.5 M citrate, 25 mM potassium acetate, 0.5 mg/mL BSA, 5 mM ADPGlc, 5 mM 2-mercaptoethanol, and 20 mg/mL glycogen) for 36 hours at 30°C. The gel was stained with iodine solution (0.2% iodine and 2% potassium iodide in 10 mM HCl) and photographed on a light box.

### Starch Granule Isolation

Starch was isolated using a small-scale wet milling procedure [46]. Maize kernels (5g), were covered with 0.45% [w/v] Na_2_S_2_O_5_ solution to about 2.5 cm above the kernels and were incubated in a 50°C water bath for 24 hours. The pericarp and embryo were removed and the endosperms were homogenized in 10 mL of 50 mM NaCl solution for 2 min with an IKA T-18 Basic Ultra Turrax Homogenizer (Cole-Parmer, Vernon Hills, IL). The homogenate was filtered through 2 layers of cheesecloth to remove large debris and washed with an additional 10 mL of 50 mM NaCl. The starch granules were allowed to settle at room temperature for 3 hours. Then the supernatant was removed and the starch was washed 5 times by suspending the starch in a 5:1 ratio of 50 mM NaCl: Toluene (10 mL NaCl and 2 mL Toluene), followed by centrifugation at 1,900 g in a clinical centrifuge (VWR, Radnor, PA) and the supernatant was discarded. The starch was washed once with 10 mL H_2_O followed by 10 mL acetone. The purified starch was dried in a fume hood for 72 hours. The purified, dried starch was stored at room temperature before use.

### Differential Scanning Calorimetry

Purified starch from 3 independent ears of each line was suspended in a 1:3 slurry with H_2_O and transferred to a sample pan and weighed. The mass of suspended starch was recorded and the sample pan was sealed in a crimping device. The sample pan and a reference pan were placed on the sample pedestals of a differential scanning calorimeter (Q200, TA Instruments, New Castle, DE). Scans were performed using a heating rate of 5°C/min from 35–95°C. The onset, peak endotherm and total enthalpy of melting were determined using the DSC built-in analysis software (TA Instruments, New Castle, DE). Statistical analysis of the data was performed using JMP (Version Pro 9.0, SAS Institute Inc., Cary, NC).

### Measurements of Kernel Vitreousness

Kernel vitreousness was measured by digital image analysis as described previously [16]. Kernels were mounted over holes in a black card stock mask and placed over a light box. Digital images were acquired using a digital camera with fixed shutter, aperture and ISO exposure settings, which were empirically determined to be below saturation for very vitreous kernels. The images were imported into Photoshop (Adobe Inc., San Jose, CA) or NIH ImageJ (NIH, Bethesda, MD) and the freehand selection tool was used to outline the endosperm. Average pixel intensity was recorded in arbitrary units.

### Glucan Chain Length Distribution

The distribution of glucan chain length was determined by fluorescence-assisted capillary electrophoresis (FACE) as described previously [47]. Starch was solubilized by boiling 4 mg in 200 μl 100% dimethyl sulfoxide. Then 2 μl of the solubilized starch was mixed with 38 μl 500 mM sodium acetate pH 4.4, and debranched by incubating with 2 μL of Pseudomonas isoamylase (Megazyme International. Wicklow, Ireland) at 42°C overnight. Then 10 μl of the debranched starch was dried in Eppendorf Vacufuge vacuum concentrator (Eppendorf, Hauppauge, NY) at 30°C for 1 hour. Then 2 μL of 1 M sodium cyanoborohydride (Sigma-Aldrich Co., St. Louis, MO) and 2 μL of 10% [w/v] 8-amino-1,3,6-pyrenetrisulfonic acid (APTS; Biotium, Inc., Scarborough, ON, Canada) solution made by resuspending 10 mg powdered APTS in 96 μL of 15% [v/v] acetic acid. The samples were separated on a Beckman P/ACE capillary electrophoresis instrument equipped with a laser activated fluorescence detector. The analysis software was used to extract percent peak area as a measure of the relative abundance of glucan chains.

### Statistical Tests and Calculation of Polydispersity Index

The starch thermal properties, and average chain length were tested by each pair *t* test with the significance level p<0.05. Pullulanase activity and polydispersity index (PDI) for W64A+, W64A*o2* and K0326Y were tested by all pairs Tukey HSD analysis with a significance level of p<0.05. All correlation data was tested by ANOVA of the slope with the significance level of p<0.05. All above tests were performed using JMP (Version 9.0, SAS Institute Inc., Cary, NC) or SPSS (Version 16.0.0, SPSS Inc., Armonk, New York). Nonlinear regression analysis of PDI and kernel vitreousness was performed using SAS 9.2 (SAS Institute Inc. Cary, NC) with 95% confidence interval of Growth Rate.

PDI was more precisely defined as degree-of-polymerization dispersity, *Đ*_*X*_ [48], which is a quantitative measurement to reflect the characteristic of degree-of-polymerization distribution. PDI was calculated according to Eq 1:

$${Đ}_{X}=\frac{{\bar{X}}_{W}}{{\bar{X}}_{n}}=\frac{\sum (\frac{A\times DP}{\sum \left(A\times DP\right)}\times DP)}{\frac{\sum \left(A\times DP\right)}{\sum A}}$$ (1)

Where *Đ*_*X*_ is the ratio of the mass-average degree of polymerization, ${\bar{X}}_{W}$, to the number-average degree of polymerization, ${\bar{X}}_{n}$, and *DP* represents the degree of polymerization of each linear glucan chain, and *A* represents the percent area, which demonstrates the frequency, corresponding to each linear glucan chain. ${\bar{X}}_{W}$ is expressed by the sum of product of weight fraction of each linear glucan chain *Đ*_*X*_ was described as the ratio of the mass-average degree of polymerization, ${\bar{X}}_{W}$, Eq 2:

$$\sum (\frac{A\times DP}{\sum \left(A\times DP\right)}\times DP)$$ (2)

and the number-average degree of polymerization, ${\bar{X}}_{n}$, which is the ratio of total degree of polymerization to total percent area, Eq 3:

$$\frac{\sum \left(A\times DP\right)}{\sum A}$$ (3)

### Scanning Electron Microscopy

Mature maize kernels were cut medial-longitudinally with a razor blade and mounted on SEM pedestals with double-sided carbon tapes. After sputter-coating with gold, the samples were observed with a JEOL JSM5410 scanning electron microscope at 25mm working distance and 10kV beam acceleration.

## Supporting Information

S1 Fig Complete multiple sequence alignment of the SSIII coding region nucleotide sequences. Nucleotide differences with yellow backgrounds represent codon changes that alter the amino acid sequence and nucleotide differences with gray backgrounds represent silent SNPs.
(PDF)

S2 Fig Complete multiple sequence alignment of the SSIII coding region protein sequences. Amino acid differences are highlighted with a yellow background.
(PDF)

S3 Fig Correlation between kernel density and hardness and pullulanase activity. (A) No significant correlation was found between kernel density and pullulanase activity, tested by ANOVA of slope (*p*>0.05). (B) No significant correlation was found between kernel hardness and pullulanase activity tested by ANOVA of slope (*p*>0.05).
(PDF)

S4 Fig Full gel images of SSIII abundance and SSIII activity of W64A+, W64A*o2*, K0326Y and *du*1M4. (PDF)

S5 Fig Full gel images for western blot of SSIII among RILs homologous for W64A*o2* (W) or QPM (Q)–derived *Zpu1* or *SSIII* alleles. (A) Western blot with SSIII antiserum. (B) Western blot with anti-actin monoclonal antibody.
(PDF)

S6 Fig Full SDS-PAGE gel image of crude protein extracts from endosperms of parent lines, W64A+, W64Ao2 and K0326Y. (PDF)

S7 Fig Full SDS-PAGE gel image of crude protein extracts from endosperms of RILs. (PDF)

S1 Table Primers used to sequence the *Zpu1* gene. (DOCX)
