# Supplementary material for: Pullulanase and Starch Synthase III Are Associated with Formation of Vitreous Endosperm in Quality Protein Maize
Source: PLoS One. 2015 Jun 26;10(6):e0130856. doi: 10.1371/journal.pone.0130856 (PMC4482715; doi:10.1371/journal.pone.0130856)
Supplement: S1 Fig — Nucleotide differences with yellow backgrounds represent codon changes that alter the amino acid sequence and nucleotide differences with gray backgrounds represent silent SNPs. (PDF) [file pone.0130856.s001.pdf]

S1 Fig. Complete multiple sequence alignment of the SSIII coding region nucleotide sequences. Nucleotide differences with yellow backgrounds represent codon changes that alter the amino acid sequence and nucleotide differences with with gray backgrounds represent silent SNPs.

```
          1        10        20        30        40        50        60
          |         |         |         |         |         |         |
 W64A+    ATGGAGATGGTCCTACGGTCGCAGAGCCCTCTATGCCTTCGGAGTGGGCCGGTGCTCATT
W64Ao2    ATGGAGATGGTCCTACGGTCGCAGAGCCCTCTATGCCTTCGGAGTGGGCCGGTGCTCATT
K0326Y    ATGGAGATGGTCCTACGGTCGCAGAGCCCTCTCTGCCTTCGGAGTGGGCCGGTGCTCATT

 W64A+    TTTCGGCCAACCGTCGCGGGCGGAGGAGGGGGCACTCAGTCTTTGTTGAGGACTACCAGA
W64Ao2    TTTCGGCCAACCGTCGCGGGCGGAGGAGGGGGCACTCAGTCTTTGTTGAGGACTACCAGA
K0326Y    TTTCGACCAACCGTCGCGGGCGGAGGAGGGGGCACTCAGTCTTTGTTGAGGACTACCAGA

 W64A+    TTTGCGAGAAGAAGGGTCATTCGATGCGTTGTAGCAAGTCCAGGTTGTCCTAATAGGAAA
W64Ao2    TTTGCGAGAAGAAGGGTCATTCGATGCGTTGTAGCAAGTCTAGGTTGTCCTAATAGGAAA
K0326Y    TTTGCGAGAAGAAGGGTCATTCGATGCGTTGTAGCAAGTCCAGGTTGTCCTAATAGGAAA

 W64A+    TCTAGGACAGCGTCTCCCAACGTAAAAGTAGCTGCTTATAGCAACTATGCGCCAAGACTC
W64Ao2    TCTAGGACAGCGTCTCCCAACGTAAAAGTAGCTGCTTATAGCAACTATGCGCCAAGACTC
K0326Y    TCTAGGACAGCGTCTCCCAACGTAAAAGTAGCTGCTTATAGCAACTATGCGCCAAGACTC

 W64A+    CTCGTTGAGTCAAGCTCCAAGAAGAGCGAACACCATGATAGCAGCAGACACCGTGAAGAA
W64Ao2    CTCGTTGAGTCAAGCTCCAAGAAGAGCGAACACCATGATAGCAGCAGACACCGTGAAGAA
K0326Y    CTCGTTGAGTCAAGCTCCAAGAAGAGCGAACACCATGATAGCAGCAGACACCGTGAAGAA

 W64A+    ACTATTGATACATACAATGGGCTGTCAGGTTCTGATGCAGCAGAATTGACAAGTAATAGA
W64Ao2    ACTATTGATACATACAATGGGCTGTCAGGTTCTGATGCAGCAGAATTGACAAGTAATAGA
K0326Y    ACTATTGATACATACAATGGGCTGTCAGGTTCTGATGCAGCAGAATTGACAAGTAATAGA

 W64A+    GATGTAGAAATTGAAGTGGATTTGCAGCACATTTCTGAGGAGGAATTGCCAGGAAAAGTA
W64Ao2    GATGTAGAAATTGAAGTGGATTTGCAGCACATTTCTGAGGAGGAATTGCCAGGAAAAGTA
K0326Y    GATGTAGAAATTGAAGTGGATTTGCAGCACATTTCTGAGGAGGAATTGCCAGGAAAAGTA

 W64A+    TCGATTAATGCATCATTAGGAGAAATGGAAACAGTGGATGAAGCTGAGGTCGAGGAGGAT
W64Ao2    TCGATTAATGCATCATTAGGAGAAATGGAAACAGTGGATGAAGCTGAGGTCGAGGAGGAT
K0326Y    TCGATTAATGCATCATTAGGAGAAATGGAAACAGTGGATGAAGCTGAGGTCGAGGAGGAT

 W64A+    AAGTTTGAGGTAGATACCTCAGGAATTGTATTGCGCAATGCTGCAGTTCGGGAAGTGGAT
W64Ao2    AAGTTTGAGGTAGATACCTCAGGAATTGTATTGCGCAATGCTGCAGTTCGGGAAGTGGAT
K0326Y    AAGTTTGAGGTAGATACCTCAGGAATTGTATTGCGCAATGTTGCAGTTCGGGAAGTGGAT

 W64A+    CCAAAGGATGAACATAATGCTAAAGATGTATTTGTGGTAGATTCGTCAGGAACTGCACCA
W64Ao2    CCAAAGGATGAACATAATGCTAAAGATGTATTTGTGGTAGATTCGTCAGGAACTGCACCA
K0326Y    CCAAAGGATGAACATAATGCTAAAGATGTATTTGTGGTAGATTCGTCAGGAACTGCACCA

 W64A+    GATAATGCTGCAGTGGAGGAAGTGGTAGATGAAGCTGAGGTTGAAGAGGATATGGTTGAT
W64Ao2    GATAATGCTGCAGTGGAGGAAGTGGTAGATGAAGCTGAGGTTGAAGAGGATATGGTTGAT
K0326Y    GATAATGCTGCAGTGGAGGAAGTGGTAGATGAAGCTGAGGTTGAAGAGGATATGGTTGAT

 W64A+    GTGGATATCTTGGGACTTGACTTGAATAATGCAACGATGGAGGAAATTGATTTGATGGAA
W64Ao2    GTGGATATCTTGGGACTTGACTTGAATAATGCAACGATGGAGGAAATTGATTTGATGGAA
K0326Y    GTGGATATCTTGGGACTTGACTTGAATAATGCAACGATCGAGGAAATTGATTTGATGGAA
```

```
 W64A+    GAGGCTTTACTGGAGAATTTCGACGTGGATTCACCAGGCAATGCTTCTAGTGGTCGAACC
W64Ao2    GAGGCTTTACTGGAGAATTTCGACGTGGATTCACCAGGCAATGCTTCTAGTGGTCGAACC
K0326Y    GAGGCTTTACTGGAGAACTTCGACGTGGATTCACCAGGCAATGCTTCTAGTGGTCGAACC

 W64A+    TATGGGGGTGTGGATGAGTTGGGTGAGCTGCCTTCAACATCCGTGGATTGCATCGCCATT
W64Ao2    TATGGGGGTGTGGATGAGTTGGGTGAGCTGCCTTCAACATCCGTGGATTGCATCGCCATT
K0326Y    TATGGGGGTGTGGATGAGTTGGGTGAGCTGCCTTCAACATCCGTGGATTGCATCGCCATT

 W64A+    AACGGAAAACATAGAAGTTTGAAGCCTGAGCCCTTGCCAATTGTCAGGTTCCAGGAACAA
W64Ao2    AACGGAAAACATAGAAGTTTGAAGCCTGAGCCCTTGCCAATTGTCAGGTTCCAGGAACAA
K0326Y    AACGGAAAACGTAGAAGTTTGAAGCCTAAGCCCTTGCCAATTGTCAGGTTCCAGGAACAA

 W64A+    GAACAGATAGTTTTAAGCATTGTTGACGAAGAAGGGTTGATTGCTGGTTCATGTGAAGAA
W64Ao2    GAACAGATAGTTTTAAGCATTGTTGACGAAGAAGGGTTGATTGCTGGTTCATGTGAAGAA
K0326Y    GAACAGATAGTTTTAAGCATTGTTGACGAAGAAGGGTTGATTGCTAGTTCATGTGAAGAA

 W64A+    GGCCAACCGGTGGTAGATTACGATAAGCAAGAGGAAAACTCTACAGCTTTCGATGAACAG
W64Ao2    GGCCAACCGGTGGTAGATTACGATAAGCAAGAGGAAAACTCTACAGCTTTCGATGAACAG
K0326Y    GGCCAACCGGTGGTAGATTACGATAAGCAAGAGGAAAACTCTACCGCTTTCGATGAACAG

 W64A+    AAGCAATTAACTGACGATTTCCCTGAAGAAGGCATATCTATAGTTCACTTCCCTGAGCCA
W64Ao2    AAGCAATTAACTGACGATTTCCCTGAAGAAGGCATATCTATAGTTCACTTCCCTGAGCCA
K0326Y    AAGCAATTAACTGATGATTTCCCTGAAGAAGGCATATCTATAGTTCACTTCCCTGAGCCA

 W64A+    AACAATGATATTGTTGGATCCTCAAAATTCTTGGAGCAAAAACAAGAATTGGATGGTTCT
W64Ao2    AACAATGATATTGTTGGATCCTCAAAATTCTTGGAGCAAAAACAAGAATTGGATGGTTCT
K0326Y    AACAATGATATTGTTGGATCCTCAAAATTCTTGGAGCAAAAACAAGAATTGGATGGTTCT

 W64A+    TATAAACAAGATCGATCAACCACTGGATTGCATGAACAAGATCAGTCTGTTGTTAGTTCA
W64Ao2    TATAAACAAGATCGATCAACCACTGGATTGCATGAACAAGATCAGTCTGTTGTTAGTTCA
K0326Y    TATAAACAAGATCGATCAACCACTGGATTGCATGAACAAGATCAGTCTGTTGTTAGTTCA

 W64A+    CATGGACAAGATAAATCAATTGTTGGTGTGCCTCAGCAAATCCAGTACAATGATCAATCT
W64Ao2    CATGGACAAGATAAATCAATTGTTGGTGTGCCTCAGCAAATCCAGTACAATGATCAATCT
K0326Y    CACGGACAAGATAAATCAATTGTTGGTGTGCCTCAGCAAATCCAGTACAATGATCAATCT

 W64A+    ATTGCTGGTTCTCATAGACAAGATCAATCAATTGCCGGTGCACCTGAGCAAATCCAATCT
W64Ao2    ATTGCTGGTTCTCATAGACAAGATCAATCAATTGCCGGTGCACCTGAGCAAATCCAATCT
K0326Y    ATTGCTGGTTCTCATAGACAAGATCAATCAATTGCCGGTGCACCTGAGCAAATCCAATCC

 W64A+    GTTGCTGGCTTTATAAAACCAAATCAATCTATTGTTGGTTCTTATAAACAACATGAATTG
W64Ao2    GTTGCTGGCTTTATAAAACCAAATCAATCTATTGTTGGTTCTTATAAACAACATGAATTG
K0326Y    GTTGCTGGCTATATAAAACCAAATCAATCTATTGTTGGTTCTTGTAAACAACATGAATTG

 W64A+    ATTATTCCTGAGCCTAAGAAAATCGAATCCATCATCAGTTACAATGAAATAGATCAATCT
W64Ao2    ATTATTCCTGAGCCTAAGAAAATCGAATCCATCATCAGTTACAATGAAATAGATCAATCT
K0326Y    ATTATTCCTGAGCCTAAGAAAATCGAATCCATCATCAGTTACAATGAAATAGATCAATCT

 W64A+    ATTGTTGGTTCTCACAAACAAGACAAATCTGTTGTTAGTGTGCCTGAGCAAATCCAATCC
W64Ao2    ATTGTTGGTTCTCACAAACAAGACAAATCTGTTGTTAGTGTGCCTGAGCAAATCCAATCC
K0326Y    ATTGTTGGTTCTCACAAACAAGACAAATCTGTTGTTAGTGTGCCTGAGCAAATCCAATCC

 W64A+    ATTGTTAGTCACAGCAAACCAAATCAATCTACTATTGATTCTTATAGACAAGCTGAATCA
W64Ao2    ATTGTTAGTCACAGCAAACCAAATCAATCTACTATTGATTCTTATAGACAAGCTGAATCA
K0326Y    ATTGTTAGTCACAGCAAACCAAATCAATCTACTGTTGATTCTTATAGACAAGCTGAATCA
```

```
 W64A+    ATTATTGGTGTGCCTGAGAAAGTCCAATCCATCACCAGTTACAATAAACTAGACCAATCC
W64Ao2    ATTATTGGTGTGCCTGAGAAAGTCCAATCCATCACCAGTTACAATAAACTAGACCAATCC
K0326Y    ATTATTGGTGTGCCTGAGAAAGTCCAATCCATCACCAGTTACGATAAACTAGACCAGTCC

 W64A+    ATCGTTGGTTCTCTTAAACAAGATGAGCCTATTATTAGCGTGCATGAGAAAATTCAATCC
W64Ao2    ATCGTTGGTTCTCTTAAACAAGATGAGCCTATTATTAGCGTGCATGAGAAAATTCAATCC
K0326Y    ATTGTTGGTTCTCTTAAACAAGATGAGCCTATTATTAGCGTGCCTGAGAAAATCCAATCC

 W64A+    ATTGTCCATTACACAAAACCAAATCAGTCTATTGTTGGCTTGCCCAAACAGCAACAATCA
W64Ao2    ATTGTCCATTACACAAAACCAAATCAGTCTATTGTTGGCTTGCCCAAACAGCAACAATCA
K0326Y    ATTGTCCATTACACTAAACCAAATCAGTCTATTGTTGGCTTGCCCAAACAACAACAATCA

 W64A+    ATTGTTCATATCGTTGAACCAAAACAGTCCATAGATGGTTTCCCTAAACAAGATCTATCA
W64Ao2    ATTGTTCATATCGTTGAACCAAAACAGTCCATAGATGGTTTCCCTAAACAAGATCTATCA
K0326Y    ATTGTTCATATCGTTGAACCAAAACAGTCCATAGATGGTTTCCCTAAACAAGATCTATCA

 W64A+    ATCGTTGGAATCTCCAATGAGTTTCAAACAAAGCAACTGGCTACTGTTGGGACTCATGAT
W64Ao2    ATCGTTGGAATCTCCAATGAGTTTCAAACAAAGCAACTGGCTACTGTTGGGACTCATGAT
K0326Y    ATCGTTGGTATCTCCAATGAGTTTCAAACAAAGCAACTGGCTACTGTTGGGACTCATGAT

 W64A+    GGATTGCTTATGAAGGGTGTGGAAGCTAAGGAGACATCTCAAAAGACTGAAGGGGATACA
W64Ao2    GGATTGCTTATGAAGGGTGTGGAAGCTAAGGAGACATCTCAAAAGATTGAAGGGGATACA
K0326Y    GGATTGCTTATGAAGGGTGTGGAAGCTAAGGAGACATCTCAAAAGACTGAAGGGGATACA

 W64A+    CTTCAGGCAACGTTCAATGCCGACAACTTGTCACAGAAACATGAGGAAGGCTTAACTAAA
W64Ao2    CTTCAGGCAACGTTCAATGCCGACAACTTGTCACAGAAACATGAGGAAGGCTTAACTAAA
K0326Y    CTTCAGGCAACGTTCAATGTCGACAACTTGTCACAGAAAC---AGGAAGGCTTAACTAAA

 W64A+    GAAGCAGACGAGATAACAATTATTGAGAAAATCAATGATGAAGACCTTGTGATGATTGAA
W64Ao2    GAAGCAGACGAGATAACAATTATTGAGAAAATCAATGATGAAGACCTTGTGATGATTGAA
K0326Y    GAAGCAGACGAGATAACAATTATTGAGAAAATCAATGATGAAGACCTTGTGATGATTGAA

 W64A+    GAACAGAAAAGCATAGCCATGAATGAAGAACAGACGATTGTTACTGAAGAAGACATTCTA
W64Ao2    GAACAGAAAAGCATAGCCATGAATGAAGAACAGACGATTGTTACTGAAGAAGACATTCTA
K0326Y    GAACAGAAAAGCATAGCCATGAATGAAGAACAGACGATTGTTACCGAAGAAGACATTCCA

 W64A+    ATGGCTAAGGTTGAGATAGGAATTGACAAGGCCAAATTTTTACATCTGCTTTCTGAAGAA
W64Ao2    ATGGCTAAGGTTGAGATAGGAATTGACAAGGCCAAATTTTTACATCTGCTTTCTGAAGAA
K0326Y    ATGGCTAAGGTTGAGATAGGAATTGACAAGGCCAAATTTTTACATCTGCTTTCTGAAGAA

 W64A+    GAGAGTTCATGGGATGAAAATGAAGTGGGAATAATTGAGGCTGATGAACAGTATGAAGTC
W64Ao2    GAGAGTTCATGGGATGAAAATGAAGTGGGAATAATTGAGGCTGATGAACAGTATGAAGTC
K0326Y    GAGAGTTCATGGGATGAAAATGAAGTGGGAATAATTGAGGCTGATGAACAGTATGAAGTC

 W64A+    GATGAGACATCTATGTCCACTGAACAAGATATCCAGGAATCACCTAATGATGATTTGGAT
W64Ao2    GATGAGACATCTATGTCCACTGAACAAGATATCCAGGAATCACCTAATGATGATTTGGAT
K0326Y    GATGAGACATCTATGTCCACTGAACAAGATATCCAGGAATCACCTAATGATGATTTGGAT

 W64A+    CCACAAGCACTACAGAGTATGCTTCAAGAGCTTGCTGAAAAAAATTATTCGCTGGGAAAC
W64Ao2    CCACAAGCACTACAGAGTATGCTTCAAGAGCTTGCTGAAAAAAATTATTCGCTGGGAAAC
K0326Y    CCACAAGCACTATGGAGTATGCTTCAAGAGCTTGCTGAAAAAAATTATTCGCTGGGAAAC

 W64A+    AAGTTGTTTACTTATCCAGATGTATTGAAAGCTGATTCAACAATTGATCTCTATTTCAAT
W64Ao2    AAGTTGTTTACTTATCCAGATGTATTGAAAGCTGATTCAACAATTGATCTCTATTTCAAT
K0326Y    AAGTTGTTTACTTATCCAGATGTATTGAAAGCTGATTCAACAATTGATCTCTATTTCAAT
```

```
 W64A+    CGTGATCTATCAGCTGTGGCCAATGAGCCTGATGTACTTATCAAAGGAGCATTCAATGGG
W64Ao2    CGTGATCTATCAGCTGTGGCCAATGAGCCTGATGTACTTATCAAAGGAGCATTCAATGGG
K0326Y    CGTGATCTATCAGCTGTGGCCAATGAGCCTGATGTACTTATCAAAGGAGCATTCAATGGG

 W64A+    TGGAAGTGGAGATTTTTCACTGAAAAATTGCACAAGAGCGAGCTGGCAGGGGACTGGTGG
W64Ao2    TGGAAGTGGAGATTTTTCACTGAAAAATTGCACAAGAGCGAGCTGGCAGGGGACTGGTGG
K0326Y    TGGAAGTGGAGATTTTTCACTGAAAAATTGCACAAGAGCGAGCTGGCAGGGGACTGGTGG

 W64A+    TGCTGCAAACTATACATTCCTAAGCAGGCATACAGAATGGACTTTGTGTTTTTTAACGGA
W64Ao2    TGCTGCAAACTATACATTCCTAAGCAGGCATACAGAATGGACTTTGTGTTTTTTAACGGA
K0326Y    TGCTGCAAACTATACATTCCTAAGCAGGCATACAGAATGGACTTTGTGTTTTTTAACGGA

 W64A+    CGCACGATATATGAAAATAATGACAATAATGATTTCGTGATACAAATAGAAAGCACCATG
W64Ao2    CGCACGATATATGAAAATAATGACAATAATGATTTCGTGATACAAATAGAAAGCACCATG
K0326Y    CACACGGTATATGAAAATAATAACAATAATGATTTCGTGATACAAATAGAAAGCACCATG

 W64A+    GATGAAAATTTATTTGAGGATTTCTTGGCTGAAGAAAAGCAACGAGAACTTGAGAACCTT
W64Ao2    GATGAAAATTTATTTGAGGATTTCTTGGCTGAAGAAAAGCAACGAGAACTTGAGAACCTT
K0326Y    GATGAAAATTTATTTGAGGATTTCTTGGCTGAAGAAAAGCAACGAGAACTTGAGAACCTT

 W64A+    GCAAATGAGGAAGCTGAAAGGAGGAGACAAACTGATGAGCAGCGGCGAATGGAGGAAGAA
W64Ao2    GCAAATGAGGAAGCTGAAAGGAGGAGACAAACTGATGAGCAGCGGCGAATGGAGGAAGAA
K0326Y    GCAAATGAGGAAGCTGAAAGGAGGAGACAAACTGATGAGCAGCGGCGAATGGAGGAAGAA

 W64A+    AGGGCCGCAGATAAAGCTGACAGGGTACAAGCCAAGGTTGAGGTAGAAACAAAGAAGAAT
W64Ao2    AGGGCCGCAGATAAAGCTGACAGGGTACAAGCCAAGGTTGAGGTAGAAACAAAGAAGAAT
K0326Y    AGGGCCGCAGATAAAGCTGACAGGGTACAAGCCAAGGTTGAGGTAGAGACGAAGAAGAAT

 W64A+    AAATTGTGCAATGTATTGGGTTTAGCCAGAGCCCCTGTTGATAATTTATGGTACATTGAG
W64Ao2    AAATTGCGCAATGTATTGGCTTTAGCCAGAGCCCCTGTTGATAATTTATGGTACATTGAG
K0326Y    AAATTGTGCAATGTATTGGGTTTAGCCAGAGCTCCTGTTGATAATTTATGGTACATTGAG

 W64A+    CCCATCACGACTGGACAAGAGGCTACTGTCAGATTGTATTATAACATAAACTCAAGACCT
W64Ao2    CCCATCACGACTGGACAAGAGGCTACTGTCAGATTGTATTATAACATAAACTCAAGACCT
K0326Y    CCCATCACGACTGGACAAGAGGCTACTGTCAGATTGTATTATAACATAAACTCAAGACCT

 W64A+    CTAGTTCACAGTACTGAGATATGGATGCATGGTGGCTATAACAATTGGATTGATGGACTC
W64Ao2    CTAGTTCACAGTACTGAGATATGGATGCATGGTGGCTATAACAATTGGATTGATGGACTC
K0326Y    CTAGTTCACAGTACTGAGATATGGATGCATGGTGGCTATAACAATTGGATTGATGGACTC

 W64A+    TCTTTTGCTGAAAGGCTTGTTCATCATAATGACAAAGATTGTGATTGGTGGTTTGCAGAT
W64Ao2    TCTTTTGCTGAAAGGCTTGTTCATCATAATGACAAAGATTGTGATTGGTGGTTTGCAGAT
K0326Y    TCTTTTGCTGAAAGGCTTGTTCATCATCATGACAAAGATTGTGATTGGTGGTTTGCAGAT

 W64A+    GTTGTCGTGCCTGAAAGAACATATGTGTTGGATTGGGTTTTTGCTGACGGCCCGCCAGGG
W64Ao2    GTTGTCGTGCCTGAAAGAACATATGTGTTGGATTGGGTTTTTGCTGACGGCCCGCCAGGG
K0326Y    GTTGTCGTGCCTGAAAGAACATATGTATTGGACTGGGTTTTTGCTGACGGCCCACCAGGG

 W64A+    AGTGCAAGGAATTATGACAACAATGGAGGACATGATTTTCATGCTACCCTTCCAAATAAC
W64Ao2    AGTGCAAGGAATTATGACAACAATGGAGGACATGATTTTCATGCTACCCTTCCAAATAAC
K0326Y    AGTGCAAGGAATTATGACAACAATGGAGGACATGATTTTCATGCTACCCTTCCAAATAAC

 W64A+    ATGACTGATGAAGAGTATTGGATGGAAGAAGAACAAAGGATCTATACAAGGCTTCAACAA
W64Ao2    ATGACTGATGAAGAGTATTGGATGGAAGAAGAACAAAGGATCTATACAAGGCTTCAACAA
K0326Y    ATGACTGAGGAAGAGTATTGGATGGAAGAAGAACAAAGGATCTATACAAGGCTTCAACAA
```

```
 W64A+    GAGAGGAGGGAAAGGGAGGAGGCTATTAAAAGGAAGGCTGAGAGAAATGCAAAAATGAAA
W64Ao2    GAGAGGAGGGAAAGGGAGGAGGCTATTAAAAAGAAGGCTGAGAGAAATGCAAAAATGAAA
K0326Y    GAGAGGAGGGAAAGGGAGGAGGCTATTAAAAGGAAGGCTGAGAGAAATGCAAAAATGAAA

 W64A+    GCTGAGATGAAGGAAAAGACTATGAGAATGTTCCTGGTTTCTCAGAAACACATTGTTTAC
W64Ao2    GCTGAGATGAAGGAAAAGACTATGAGAATGTTCCTGGTTTCTCAGAAACACATTGTTTAC
K0326Y    GCTGAGATGAAGGAAAAGACTATGAGAATGTTCCTGGTTTCTCAGAAACACATTGTTTAC

 W64A+    ACCGAACCACTTGAAATACATGCTGGAACTACTATTGATGTGCTTTATAATCCTTCTAAT
W64Ao2    ACCGAACCACTTGAAATACATGCTGGAACTACTATTGATGTGCTTTATAATCCTTCTAAT
K0326Y    ACCGAACCACTTGAAATACATGCTGGAACTACTATTGATGTGCTTTATAATCCTTCTAAT

 W64A+    ACAGTTCTAACTGGAAAGCCAGAGGTTTGGTTTCGATGTTCCTTTAATCGTTGGATGTAT
W64Ao2    ACAGTTCTAACTGGAAAGCCAGAGGTTTGGTTTCGATGTTCCTTTAATCGTTGGATGTAT
K0326Y    ACAGTTCTAACTGGAAAGCCAGAGGTTTGGTTTCGATGTTCATTTAATCGTTGGATGTAT

 W64A+    CCAGGTGGGGTGTTGCCACCTCAGAGGATGGTACAAGCAGAAAATGGTTCACACCTAAAA
W64Ao2    CCAGGTGGGGTGTTGCCACCTCAGAAGATGGTACAAGCAGAAAATGGTTCACACCTAAAA
K0326Y    CCAGGTGGGGTGTTGCCACCTCAGAAGATGGTACAAGCAGAAAATGGTTCACACCTAAAA

 W64A+    GCAACAGTTTACGTTCCACGAGATGCCTATATGATGGACTTCGTTTTCTCGGAGTCAGAA
W64Ao2    GCAACAGTTTACGTTCCACGAGATGCCTATATGATGGACTTCGTTTTCTCGGAGTCAGAA
K0326Y    GTAACAGTTTACGTTCCACGAGATGCCTATATGATGGACTTTGTTTTCTCGGAGTCAGAA

 W64A+    GAAGGTGGAATTTATGATAACAGAAATGGGTTAGACTATCATATTCCTGTTTTTGGGTCA
W64Ao2    GAAGGTGGAATTTATGATAACAGAAATGGGTTAGACTATCATATTCCTGTTTTTGGGTCA
K0326Y    GAAGGTGGAATTTATGATAACAGAAATGGGTTAGACTATCATATTCCTGTTTTTGGGTCA

 W64A+    ATTGCAAAGGAACCACCTATGCACATTGTCCACATTGCTGTTGAGATGGCACCAATCGCA
W64Ao2    ATTGCAAAGGAACCACCTATGCACATTGTCCACATTGCTGTTGAGATGGCACCAATCGCA
K0326Y    ATTGCAAAGGAACCACCTATGCACATTGTCCACATCGCTGTTGAGATGGCACCAATCGCA

 W64A+    AAGGTTGGAGGTCTTGGTGATGTTGTCACTAGTCTTTCACGTGCTGTGCAAGATTTAGGA
W64Ao2    AAGGTTGGAGGTCTTGGTGATGTTGTCACTAGTCTTTCACGTGCTGTGCAAGATTTAGGA
K0326Y    AAGGTTGGAGGTCTTGGTGATGTTGTCACTAGTCTTTCACGTGCTGTGCAAGATTTAGGA

 W64A+    CACAATGTGGAGGTTATTCTTCCAAAGTACGGTTGCTTGAATCTAAGCAATGTCAAGAAT
W64Ao2    CACAATGTGGAGGTTATTCTTCCAAAGTACGGTTGCTTGAATCTAAGCAATGTCAAGAAT
K0326Y    CACAATGTGGAGGTTATTCTTCCAAAGTACGGTTGCTTGAATCTAAGCAATGTCAAGAAT

 W64A+    CTACAAATCCATCAGAGTTTTTCTTGGGGTGGTTCTGAAATAAATGTGTGGCGTGGACTA
W64Ao2    CTACAAATCCATCAGAGTTTTTCTTGGGGTGGTTCTGAAATAAATGTGTGGCGTGGACTA
K0326Y    CTACACATCCATCAGAGTTTTTCTTGGGGTGGTTCTGAAATAAAAGTGTGGCGTGGACTA

 W64A+    GTCGAAGGCCTTTGTGTTTACTTCCTGGAACCTCAAAATGGGATGTTTGGAGTCGGATAT
W64Ao2    GTCGAAGGCCTTTGTGTTTACTTCCTGGAACCTCAAAATGGGATGTTTGGAGTCGGATAT
K0326Y    GTCGAAGGCCTTTGTGTTTACTTCCTGGAACCTCAAAATGGGATGTTTGGAGTCGGATAT

 W64A+    GTATATGGCAGGGACGATGACCGCCGATTTGGCTTCTTCTGTCGTTCTGCTCTAGAGTTT
W64Ao2    GTATATGGCAGGGACGATGACCGCCGATTTGGCTTCTTCTGTCGTTCTGCTCTAGAGTTT
K0326Y    GTATATGGCAGGGACGATGACCGCCGATTTGGCTTCTTCTGTCGTTCTGCTCTAGAGTTT

 W64A+    CTCCTCCAAAGTGGATCTTCTCCTAACATAATACATTGCCATGATTGGTCAAGTGCTCCT
W64Ao2    CTCCTCCAAAGTGGATCTTCTCCTAACATAATACATTGCCATGATTGGTCAAGTGCTCCT
K0326Y    CTCCTCCAAAGTGGATCTTCTCCTAACATAATACATTGCCATGATTGGTCAAGTGCTCCT
```

```
 W64A+    GTTGCCTGGCTACACAAGGAAAACTACGCGAAGTCTAGCTTGGCAAACGCACGGGTGGTA
W64Ao2    GTTGCCTGGCTACACAAGGAAAACTACGCGAAGTCTAGCTTGGCAAACGCACGGGTGGTA
K0326Y    GTTGCCTGGCTACACAAGGAAAACTACGCGAAGTCTAGCTTGGCAAATGCACGGGTGGTA

 W64A+    TTCACCATCCACAATCTTGAATTTGGAGCGCATCATATTGGCAAAGCAATGAGATATTGT
W64Ao2    TTCACCATCCACAATCTTGAATTTGGAGCGCATCATATTGGCAAAGCAATGAGATATTGT
K0326Y    TTCACCATCCACAATCTTGAATTTGGAGCGCATCATATTGGCAAAGCAATGAGATATTGT

 W64A+    GATAAAGCAACAACTGTCTCTAATACATATTCAAAGGAAGTGTCAGGTCATGGTGCCATA
W64Ao2    GATAAAGCAACAACTGTCTCTAATACATATTCAAAGGAAGTGTCAGGTCATGGTGCCATC
K0326Y    GATAAAGCCACAACTGTCTCTAATACATATTCAAAGGAAGTGTCAGGTCATGGTGCCATC

 W64A+    GTTCCTCATCCTGGGAAATTCTATGGCATTCTCAATGGAATTGATCCGGATATATGGGAT
W64Ao2    GTTCCTCATCTTGGGAAATTCTATGGCATTCTCAATGGAATTGATCCGGATATATGGGAT
K0326Y    GTTCCTCATCTTGGGAAATTCTATGGCATTCTGAATGGAATTGATCCAGATATATGGGAT

 W64A+    CCGTACAATGACAACTTTATCCCGGTCCACTACACTTGTGAGAATGTGGTTGAAGGCAAG
W64Ao2    CCGTACAATGACAACTTTATCCCGGTCCACTACACTTGTGAGAATGTGGTTGAAGGCAAG
K0326Y    CCGTACAATGACAACTTTATCCCGGTCCACTACACTTGTGAGAATGTGGTTGAAGGCAAG

 W64A+    AGGGCTGCTAAGAGGGCACTGCAGCAGAAGTTTGGGTTACAGCAAATCGATGTCCCCGTC
W64Ao2    AGGGCTGCTAAGAGGGCACTGCAGCAGAAGTTTGGGTTACAGCAAATCGATGTCCCCGTC
K0326Y    AGGGCTGCTAAGAGGGCACTGCAGCAGAAGTTTGGGTTACAGCAAATCGATGTCCCCGTC

 W64A+    GTAGGAATCGTCACTCGCCTGACAGCCCAAAAGGGGATCCACCTGATCAAGCATGCGATT
W64Ao2    GTAGGAATCGTCACTCGCCTGACAGCCCAAAAGGGGATCCACCTGATCAAGCATGCGATT
K0326Y    GTAGGAATCGTCACTCGCCTGACAGCCCAAAAGGGGATCCACCTGATCAAGCATGCGATT

 W64A+    CACCGTACACTCGAACGGAACGGACAGGTGGTTTTGCTTGGTTCAGCGCCGGACTCTCGA
W64Ao2    CACCGTACACTCGAACGGAACGGACAGGTGGTTTTGCTTGGTTCAGCGCCGGACTCTCGA
K0326Y    CACCGTACACTCGAACGGAACGGACAGGTGGTTTTGCTTGGTTCAGCGCCGGACTCTCGA

 W64A+    ATCCAAGCTGATTTTGTCAACCTGGCGAATACGCTCCACGGCGTAAACCATGGGCAAGTG
W64Ao2    ATCCAAGCTGATTTTGTCAACCTGGCGAATACGCTCCACGGCGTAAACCATGGGCAAGTG
K0326Y    ATCCAAGCTGATTTTGTCAACCTGGCGAATAAGCTCCACGGCGTAAACCATGGGCAAGTG

 W64A+    AGGCTTTCCTTGACCTACGACGAGCCTCTCTCGCATCTGATATACGCTGGCTCTGACTTC
W64Ao2    AGGCTGTCCTTGACCTACGACGAGCCTCTCTCGCATCTGATATACGCTGGCTCTGACTTC
K0326Y    AGGCTTTCCTTGACCTACGACGAGCCTCTCTCGCATCTGATATACGCTGGCTCTGACTTC

 W64A+    ATTCTGGTCCCATCTATATTTGAGCCTTGCGGCCTAACTCAGCTCGTCGCCATGCGGTAT
W64Ao2    ATTCTGGTCCCATCTATATTTGAGCCTTGCGGCCTAACTCAGCTGGTCGCCATGCGGTAT
K0326Y    ATTCTGGTCCCATCTATATTTGAGCCTTGCGGCCTAACTCAGCTCGTCGCCATGCGGTAT

 W64A+    GGGACCATCCCGATTGTCCGCAAGACTGGAGGGCTCTTCGACACTGTCTTCGATGTGGAC
W64Ao2    GGAACCATCCCGATTGTCCGCAAGACTGGAGGGCTCTTCGACACTGTCTTCGATGTGGAC
K0326Y    GGGACCATCCCGATTGTCCGCAAGACTGGAGGGCTCTTCGACACTGTCTTCGATGTGGAC

 W64A+    AATGACAAGGAACGAGCCCGAGATCGAGGCCTTGAGCCCAACGGGTTTAGCTTTGACGGA
W64Ao2    AATGACAAGGAACGAGCCCGAGATCGAGGCCTTGAGCCCAACGGGTTTAGCTTTGACGGA
K0326Y    AATGACAAGGAACGAGCCCGAGATCGAGGCCTTGAGCCCAACGGGTTTAGCTTTGACGGA

 W64A+    GCTGATAGCAACGGTGTTGACTACGCGCTGAACAGGGCGATCTCAGCTTGGTTCGATGCC
W64Ao2    GCTGATAGCAACGGTGTTGACTACGCCCTGAACAGGGCGATCTCAGCTTGGTTCGATGCC
K0326Y    GCTGATAGCAACGGTGTTGACTACGCGCTGAACAGGGCGATCTCAGCTTGGTTCGATGCC
```

```
 W64A+   CGGAGCTGGTTCCACTCCCTTTGCAAGAGAGTCATGGAGCAGGACTGGTCGTGGAACCGA
W64Ao2   CGGAGCTGGTTCCACTCCCTTTGCAAGAGAGTCATGGAGCAGGACTGGTCGTGGAACCGA
K0326Y   CGGAGCTGGTTCCACTCCCTTTGCAAGAGAGTCATGGAGCAGGACTGGTCGTGGAACCGA

 W64A+   CCTGCCCTCGACTACATCGAGCTCTACCGTTCAGCGTCCAAATTGTAA
W64Ao2   CCTGCCCTCGACTACATCGAGCTCTACCGTTCAGCGTCCAAATTGTAA
K0326Y   CCTGCCCTCGACTACATCGAGCTCTACCGTTCAGCGTCCAAATTGTAA
```
