# Supplementary material for: Pullulanase and Starch Synthase III Are Associated with Formation of Vitreous Endosperm in Quality Protein Maize
Source: PLoS One. 2015 Jun 26;10(6):e0130856. doi: 10.1371/journal.pone.0130856 (PMC4482715; doi:10.1371/journal.pone.0130856)
Supplement: S2 Fig — Amino acid differences are highlighted with a yellow background. (PDF) [file pone.0130856.s002.pdf]

S2 Fig. Complete multiple sequence alignment of the SSIII coding region protein sequences. Amino acid differences are highlighted with a yellow background.

```
          1         10        20        30        40        50        60
          |         |         |         |         |         |         |
 W64A+    MEMVLRSQSPLCLRSGPVLIFRPTVAGGGGGTQSLLRTTRFARRRVIRCVVASPGCPNRK
W64Ao2    MEMVLRSQSPLCLRSGPVLIFRPTVAGGGGGTQSLLRTTRFARRRVIRCVVASLGCPNRK
K0326Y    MEMVLRSQSPLCLRSGPVLIFRPTVAGGGGGTQSLLRTTRFARRRVIRCVVASPGCPNRK

 W64A+    SRTASPNVKVAAYSNYAPRLLVESSSKKSEHHDSSRHREETIDTYNGLSGSDAAELTSNR
W64Ao2    SRTASPNVKVAAYSNYAPRLLVESSSKKSEHHDSSRHREETIDTYNGLSGSDAAELTSNR
K0326Y    SRTASPNVKVAAYSNYAPRLLVESSSKKSEHHDSSRHREETIDTYNGLSGSDAAELTSNR

 W64A+    DVEIEVDLQHISEEELPGKVSINASLGEMETVDEAEVEEDKFEVDTSGIVLRNAAVREVD
W64Ao2    DVEIEVDLQHISEEELPGKVSINASLGEMETVDEAEVEEDKFEVDTSGIVLRNAAVREVD
K0326Y    DVEIEVDLQHISEEELPGKVSINASLGEMETVDEAEVEEDKFEVDTSGIVLRNVAVREVD

 W64A+    PKDEHNAKDVFVVDSSGTAPDNAAVEEVVDEAEVEEDMVDVDILGLDLNNATMEEIDLME
W64Ao2    PKDEHNAKDVFVVDSSGTAPDNAAVEEVVDEAEVEEDMVDVDILGLDLNNATMEEIDLME
K0326Y    PKDEHNAKDVFVVDSSGTAPDNAAVEEVVDEAEVEEDMVDVDILGLDLNNATIEEIDLME

 W64A+    EALLENFDVDSPGNASSGRTYGGVDELGELPSTSVDCIAINGKHRSLKPEPLPIVRFQEQ
W64Ao2    EALLENFDVDSPGNASSGRTYGGVDELGELPSTSVDCIAINGKHRSLKPEPLPIVRFQEQ
K0326Y    EALLENFDVDSPGNASSGRTYGGVDELGELPSTSVDCIAINGKRRSLKPKPLPIVRFQEQ

 W64A+    EQIVLSIVDEEGLIAGSCEEGQPVVDYDKQEENSTAFDEQKQLTDDFPEEGISIVHFPEP
W64Ao2    EQIVLSIVDEEGLIAGSCEEGQPVVDYDKQEENSTAFDEQKQLTDDFPEEGISIVHFPEP
K0326Y    EQIVLSIVDEEGLIASSCEEGQPVVDYDKQEENSTAFDEQKQLTDDFPEEGISIVHFPEP

 W64A+    NNDIVGSSKFLEQKQELDGSYKQDRSTTGLHEQDQSVVSSHGQDKSIVGVPQQIQYNDQS
W64Ao2    NNDIVGSSKFLEQKQELDGSYKQDRSTTGLHEQDQSVVSSHGQDKSIVGVPQQIQYNDQS
K0326Y    NNDIVGSSKFLEQKQELDGSYKQDRSTTGLHEQDQSVVSSHGQDKSIVGVPQQIQYNDQS

 W64A+    IAGSHRQDQSIAGAPEQIQSVAGFIKPNQSIVGSYKQHELIIPEPKKIESIISYNEIDQS
W64Ao2    IAGSHRQDQSIAGAPEQIQSVAGFIKPNQSIVGSYKQHELIIPEPKKIESIISYNEIDQS
K0326Y    IAGSHRQDQSIAGAPEQIQSVAGYIKPNQSIVGSCKQHELIIPEPKKIESIISYNEIDQS

 W64A+    IVGSHKQDKSVVSVPEQIQSIVSHSKPNQSTIDSYRQAESIIGVPEKVQSITSYNKLDQS
W64Ao2    IVGSHKQDKSVVSVPEQIQSIVSHSKPNQSTIDSYRQAESIIGVPEKVQSITSYNKLDQS
K0326Y    IVGSHKQDKSVVSVPEQIQSIVSHSKPNQSTVDSYRQAESIIGVPEKVQSITSYDKLDQS

 W64A+    IVGSLKQDEPIISVHEKIQSIVHYTKPNQSIVGLPKQQQSIVHIVEPKQSIDGFPKQDLS
W64Ao2    IVGSLKQDEPIISVHEKIQSIVHYTKPNQSIVGLPKQQQSIVHIVEPKQSIDGFPKQDLS
K0326Y    IVGSLKQDEPIISVPEKIQSIVHYTKPNQSIVGLPKQQQSIVHIVEPKQSIDGFPKQDLS

 W64A+    IVGISNEFQTKQLATVGTHDGLLMKGVEAKETSQKTEGDTLQATFNADNLSQKHEEGLTK
W64Ao2    IVGISNEFQTKQLATVGTHDGLLMKGVEAKETSQKIEGDTLQATFNADNLSQKHEEGLTK
K0326Y    IVGISNEFQTKQLATVGTHDGLLMKGVEAKETSQKTEGDTLQATFNVDNLSQK-QEGLTK

 W64A+    EADEITIIEKINDEDLVMIEEQKSIAMNEEQTIVTEEDILMAKVEIGIDKAKFLHLLSEE
W64Ao2    EADEITIIEKINDEDLVMIEEQKSIAMNEEQTIVTEEDILMAKVEIGIDKAKFLHLLSEE
K0326Y    EADEITIIEKINDEDLVMIEEQKSIAMNEEQTIVTEEDIPMAKVEIGIDKAKFLHLLSEE

 W64A+    ESSWDENEVGIIEADEQYEVDETSMSTEQDIQESPNDDLDPQALQSMLQELAEKNYSLGN
W64Ao2    ESSWDENEVGIIEADEQYEVDETSMSTEQDIQESPNDDLDPQALQSMLQELAEKNYSLGN
K0326Y    ESSWDENEVGIIEADEQYEVDETSMSTEQDIQESPNDDLDPQALWSMLQELAEKNYSLGN
```

```
 W64A+   KLFTYPDVLKADSTIDLYFNRDLSAVANEPDVLIKGAFNGWKWRFFTEKLHKSELAGDWW
W64Ao2   KLFTYPDVLKADSTIDLYFNRDLSAVANEPDVLIKGAFNGWKWRFFTEKLHKSELAGDWW
K0326Y   KLFTYPDVLKADSTIDLYFNRDLSAVANEPDVLIKGAFNGWKWRFFTEKLHKSELAGDWW

 W64A+   CCKLYIPKQAYRMDFVFFNGRTIYENNDNNDFVIQIESTMDENLFEDFLAEEKQRELENL
W64Ao2   CCKLYIPKQAYRMDFVFFNGRTIYENNDNNDFVIQIESTMDENLFEDFLAEEKQRELENL
K0326Y   CCKLYIPKQAYRMDFVFFNGHTVYENNNNNDFVIQIESTMDENLFEDFLAEEKQRELENL

 W64A+   ANEEAERRRQTDEQRRMEEERAADKADRVQAKVEVETKKNKLCNVLGLARAPVDNLWYIE
W64Ao2   ANEEAERRRQTDEQRRMEEERAADKADRVQAKVEVETKKNKLRNVLALARAPVDNLWYIE
K0326Y   ANEEAERRRQTDEQRRMEEERAADKADRVQAKVEVETKKNKLCNVLGLARAPVDNLWYIE

 W64A+   PITTGQEATVRLYYNINSRPLVHSTEIWMHGGYNNWIDGLSFAERLVHHNDKDCDWWFAD
W64Ao2   PITTGQEATVRLYYNINSRPLVHSTEIWMHGGYNNWIDGLSFAERLVHHNDKDCDWWFAD
K0326Y   PITTGQEATVRLYYNINSRPLVHSTEIWMHGGYNNWIDGLSFAERLVHHHDKDCDWWFAD

 W64A+   VVVPERTYVLDWVFADGPPGSARNYDNNGGHDFHATLPNNMTDEEYWMEEEQRIYTRLQQ
W64Ao2   VVVPERTYVLDWVFADGPPGSARNYDNNGGHDFHATLPNNMTDEEYWMEEEQRIYTRLQQ
K0326Y   VVVPERTYVLDWVFADGPPGSARNYDNNGGHDFHATLPNNMTEEEYWMEEEQRIYTRLQQ

 W64A+   ERREREEAIKRKAERNAKMKAEMKEKTMRMFLVSQKHIVYTEPLEIHAGTTIDVLYNPSN
W64Ao2   ERREREEAIKKKAERNAKMKAEMKEKTMRMFLVSQKHIVYTEPLEIHAGTTIDVLYNPSN
K0326Y   ERREREEAIKRKAERNAKMKAEMKEKTMRMFLVSQKHIVYTEPLEIHAGTTIDVLYNPSN

 W64A+   TVLTGKPEVWFRCSFNRWMYPGGVLPPQRMVQAENGSHLKATVYYVPRDAYMMDFVFSESE
W64Ao2   TVLTGKPEVWFRCSFNRWMYPGGVLPPQKMVQAENGSHLKATVYYVPRDAYMMDFVFSESE
K0326Y   TVLTGKPEVWFRCSFNRWMYPGGVLPPQKMVQAENGSHLKVTVYYVPRDAYMMDFVFSESE

 W64A+   EGGIYDNRNGLDYHIPVFGSIAKEPPMHIVHIAVEMAPIAKVGGLGDVVTSLSRAVQDLG
W64Ao2   EGGIYDNRNGLDYHIPVFGSIAKEPPMHIVHIAVEMAPIAKVGGLGDVVTSLSRAVQDLG
K0326Y   EGGIYDNRNGLDYHIPVFGSIAKEPPMHIVHIAVEMAPIAKVGGLGDVVTSLSRAVQDLG

 W64A+   HNVEVILPKYGCLNLSNVKNLQIHQSFSWGGSEINVWRGLVEGLCVYFLEPQNGMFGVGY
W64Ao2   HNVEVILPKYGCLNLSNVKNLQIHQSFSWGGSEINVWRGLVEGLCVYFLEPQNGMFGVGY
K0326Y   HNVEVILPKYGCLNLSNVKNLHIHQSFSWGGSEIKVWRGLVEGLCVYFLEPQNGMFGVGY

 W64A+   VYGRDDDRRFGFFCRSALEFLLQSGSSPNIIHCHDWSSAPVAWLHKENYAKSSLANARVV
W64Ao2   VYGRDDDRRFGFFCRSALEFLLQSGSSPNIIHCHDWSSAPVAWLHKENYAKSSLANARVV
K0326Y   VYGRDDDRRFGFFCRSALEFLLQSGSSPNIIHCHDWSSAPVAWLHKENYAKSSLANARVV

 W64A+   FTIHNLEFGAHHIGKAMRYCDKATTVSNTYSKEVSGHGAIVPHPGKFYGILNGIDPDIWD
W64Ao2   FTIHNLEFGAHHIGKAMRYCDKATTVSNTYSKEVSGHGAIVPHLGKFYGILNGIDPDIWD
K0326Y   FTIHNLEFGAHHIGKAMRYCDKATTVSNTYSKEVSGHGAIVPHLGKFYGILNGIDPDIWD

 W64A+   PYNDNFIPVHYTCENVVEGKRAAKRALQQKFGLQQIDVPVVGIVTRLTAQKGIHLIKHAI
W64Ao2   PYNDNFIPVHYTCENVVEGKRAAKRALQQKFGLQQIDVPVVGIVTRLTAQKGIHLIKHAI
K0326Y   PYNDNFIPVHYTCENVVEGKRAAKRALQQKFGLQQIDVPVVGIVTRLTAQKGIHLIKHAI

 W64A+   HRTLERNGQVVLLGSAPDSRIQADFVNLANTLHGVNHGQVRLSLTYDEPLSHLIYAGSDF
W64Ao2   HRTLERNGQVVLLGSAPDSRIQADFVNLANTLHGVNHGQVRLSLTYDEPLSHLIYAGSDF
K0326Y   HRTLERNGQVVLLGSAPDSRIQADFVNLANKLHGVNHGQVRLSLTYDEPLSHLIYAGSDF

 W64A+   ILVPSIFEPCGLTQLVAMRYGTIPIVRKTGGLFDTVFDVDNDKERARDRGLEPNGFSFDG
W64Ao2   ILVPSIFEPCGLTQLVAMRYGTIPIVRKTGGLFDTVFDVDNDKERARDRGLEPNGFSFDG
K0326Y   ILVPSIFEPCGLTQLVAMRYGTIPIVRKTGGLFDTVFDVDNDKERARDRGLEPNGFSFDG
```

```
 W64A+   ADSNGVDYALNRAISAWFDARSWFHSLCKRVMEQDWSWNRPALDYIELYRSASKL*
W64Ao2   ADSNGVDYALNRAISAWFDARSWFHSLCKRVMEQDWSWNRPALDYIELYRSASKL*
K0326Y   ADSNGVDYALNRAISAWFDARSWFHSLCKRVMEQDWSWNRPALDYIELYRSASKL*
```
