# Supplementary material for: Pullulanase and Starch Synthase III Are Associated with Formation of Vitreous Endosperm in Quality Protein Maize
Source: PLoS One. 2015 Jun 26;10(6):e0130856. doi: 10.1371/journal.pone.0130856 (PMC4482715; doi:10.1371/journal.pone.0130856)
Supplement: S3 Fig — (A) No significant correlation was found between kernel density and pullulanase activity, tested by ANOVA of slope (p>0.05). (B) No significant correlation was found between kernel hardness and pullulanase activity tested by ANOVA of slope (p>0.05). (PDF) [file pone.0130856.s003.pdf]

A

B

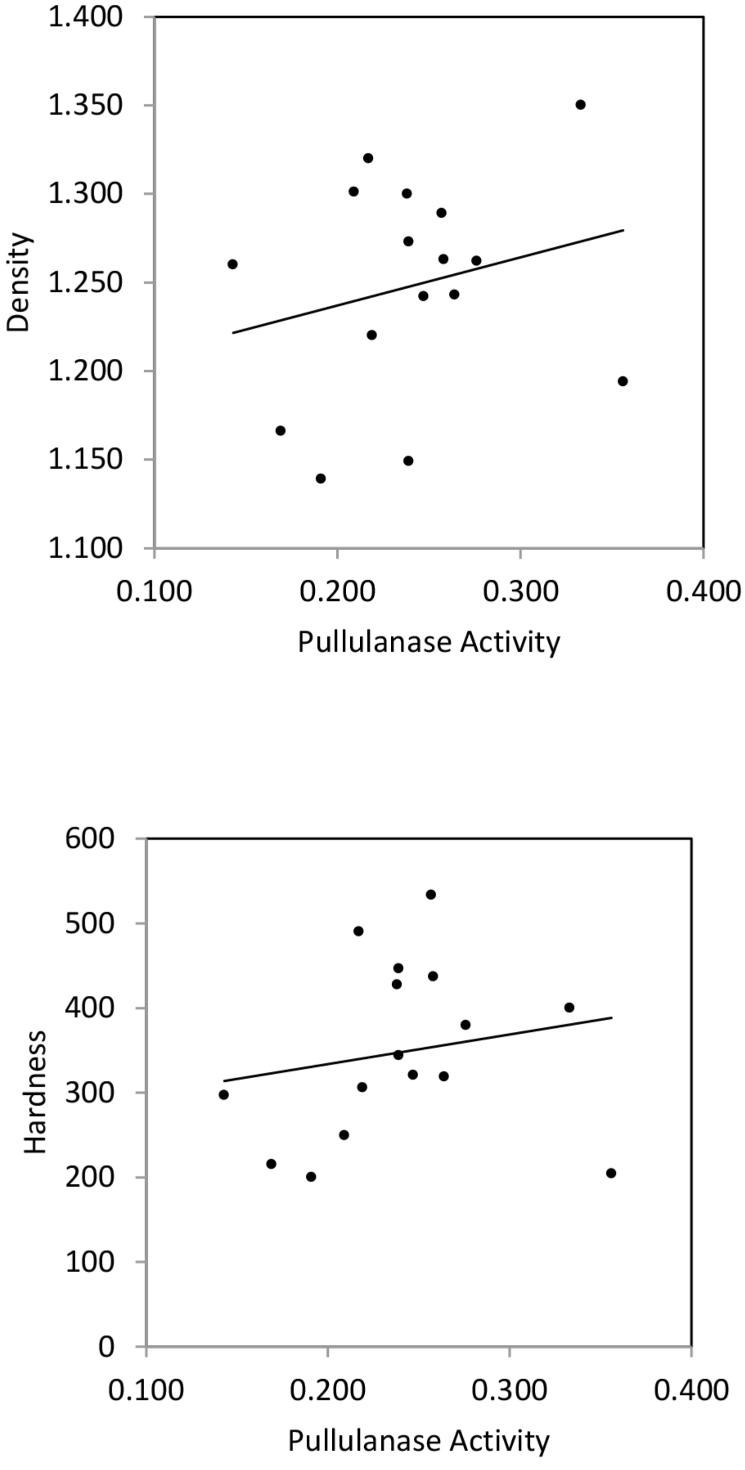

S1 Fig. Correlation between kernel density and hardness and pullulanase activity. (A) No significant correlation was found between kernel density and pullulanase activity, tested by ANOVA of slope (p>0.05). (B) No significant correlation was found between kernel hardness and pullulanase activity tested by ANOVA of slope (p>0.05).
