# Supplementary figures and images for: Pullulanase and Starch Synthase III Are Associated with Formation of Vitreous Endosperm in Quality Protein Maize
Source: PLoS One. 2015 Jun 26;10(6):e0130856. doi: 10.1371/journal.pone.0130856 (PMC4482715; doi:10.1371/journal.pone.0130856)

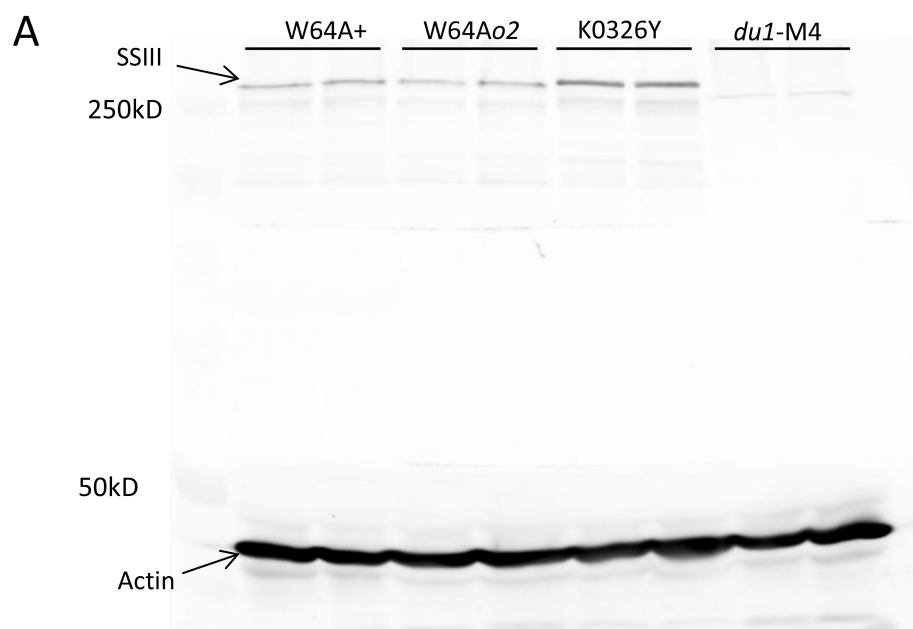

**B**

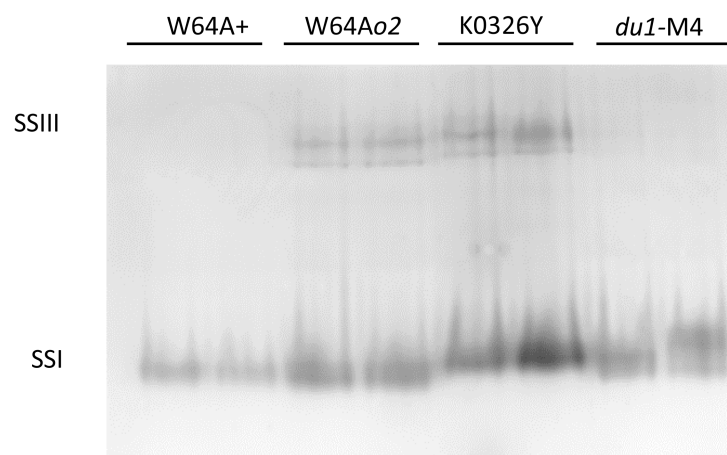

**S4 Fig. Full gel images of SSIII abundance and SSIII activity of W64A+, W64Ao2, K0326Y and *du1M4*.**

Supplement: S4 Fig — (PDF) [file pone.0130856.s004.pdf]
