# Supplementary material for: Pullulanase and Starch Synthase III Are Associated with Formation of Vitreous Endosperm in Quality Protein Maize
Source: PLoS One. 2015 Jun 26;10(6):e0130856. doi: 10.1371/journal.pone.0130856 (PMC4482715; doi:10.1371/journal.pone.0130856)
Supplement: S5 Fig — (A) Western blot with SSIII antiserum. (B) Western blot with anti-actin monoclonal antibody. (PDF) [file pone.0130856.s005.pdf]

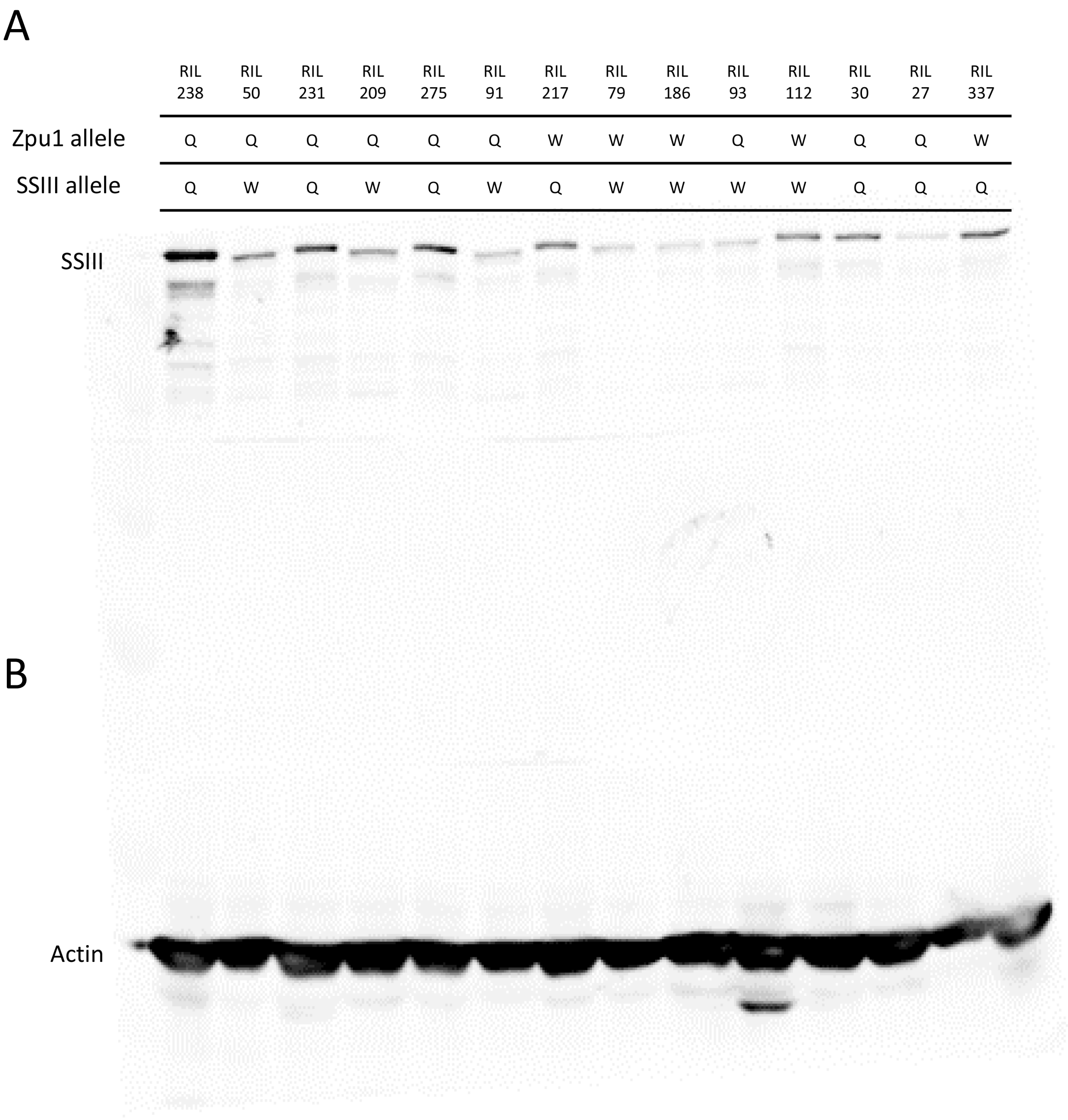

S5 Fig. Full gel images for western blot of SSIII among RILs homologous for W64A*o2* (W) or QPM (Q) – derived *Zpu1* or *SSIII* alleles. (A) Western blot with SSIII antiserum. (B) Western blot with anti-actin monoclonal antibody.
