# Supplementary material for: Pullulanase and Starch Synthase III Are Associated with Formation of Vitreous Endosperm in Quality Protein Maize
Source: PLoS One. 2015 Jun 26;10(6):e0130856. doi: 10.1371/journal.pone.0130856 (PMC4482715; doi:10.1371/journal.pone.0130856)
Supplement: S6 Fig — (PDF) [file pone.0130856.s006.pdf]

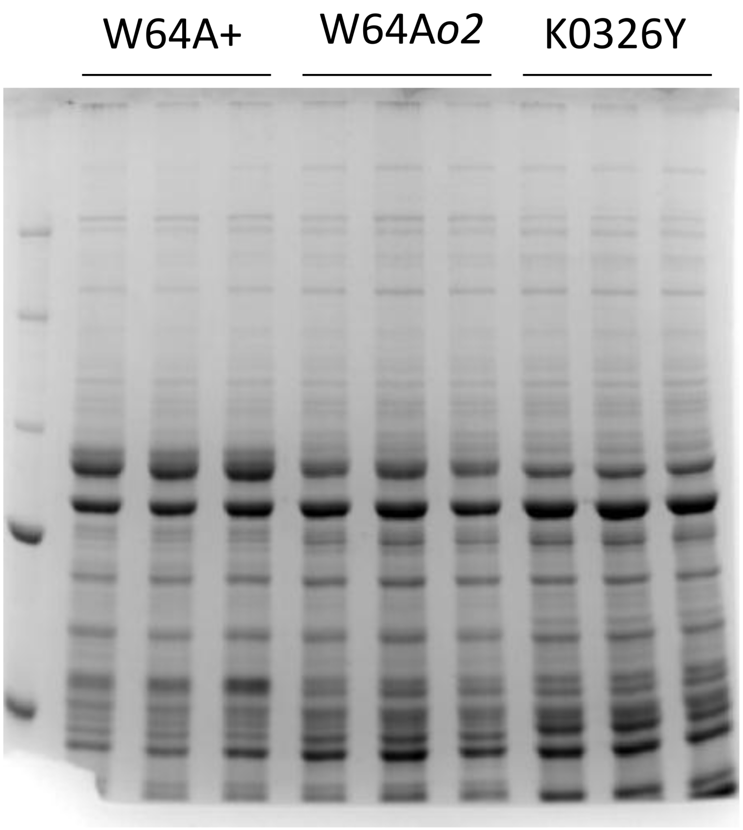

S6 Fig. Full SDS-PAGE gel image of crude protein extracts from endosperms of parent lines, W64A+, W64Ao2 and K0326Y.
