# Supplementary material for: Pullulanase and Starch Synthase III Are Associated with Formation of Vitreous Endosperm in Quality Protein Maize
Source: PLoS One. 2015 Jun 26;10(6):e0130856. doi: 10.1371/journal.pone.0130856 (PMC4482715; doi:10.1371/journal.pone.0130856)
Supplement: S7 Fig — (PDF) [file pone.0130856.s007.pdf]

	RIL 238	RIL 50	RIL 231	RIL 209	RIL 275	RIL 91	RIL 217	RIL 79	RIL 186	RIL 93	RIL 112	RIL 30	RIL 27	RIL 337
Zpu1 allele	Q	Q	Q	Q	Q	Q	W	W	W	Q	W	Q	Q	W
SSIII allele	Q	W	Q	W	Q	W	Q	W	W	W	W	Q	Q	Q

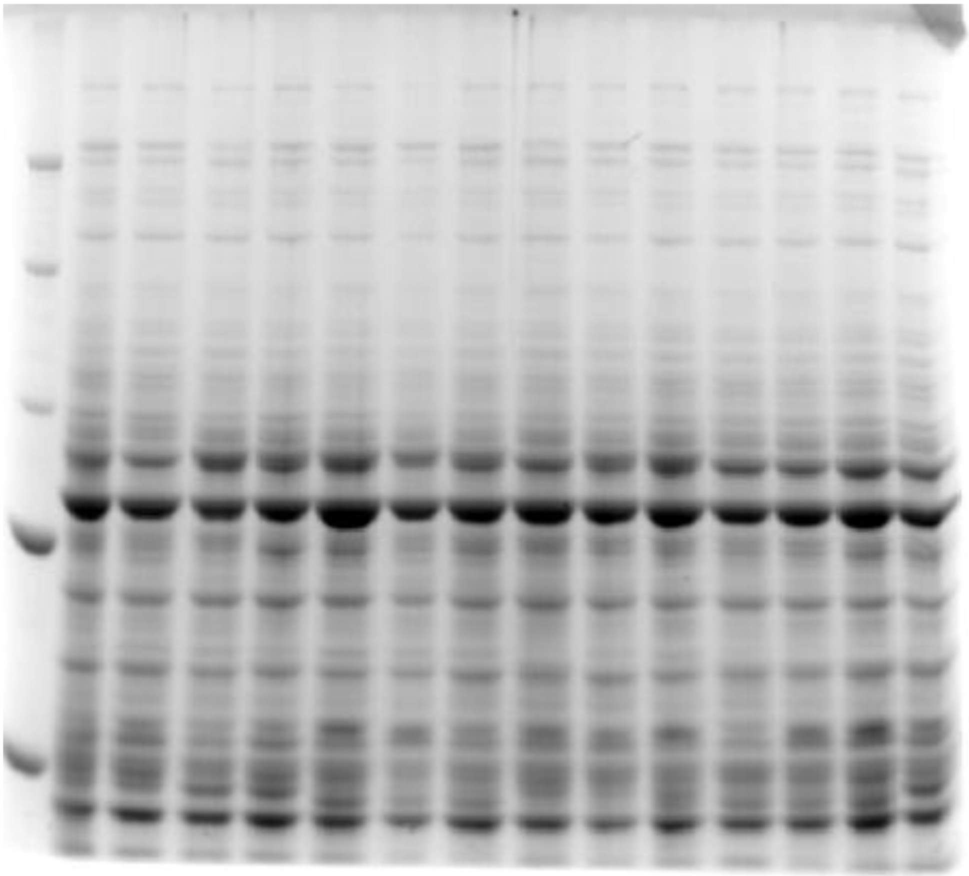

S7 Fig. Full SDS-PAGE gel image of crude protein extracts from endosperms of RILs.
