# Supplementary material for: Pullulanase and Starch Synthase III Are Associated with Formation of Vitreous Endosperm in Quality Protein Maize
Source: PLoS One. 2015 Jun 26;10(6):e0130856. doi: 10.1371/journal.pone.0130856 (PMC4482715; doi:10.1371/journal.pone.0130856)
Supplement: S1 Table — (DOCX) [file pone.0130856.s008.docx]

**S1 Table. Primers used to sequence the *Zpu1* gene.**

| **Name** | **Sequence** | **Start** | **End** | **Product Size** |
| --- | --- | --- | --- | --- |
| *Zpu1*_1F | ACCGCCTTCTCTCTCCCTCCGA | -40^a^ | 276 | 317 |
| *Zpu1*_1R | CACCCAGTAAGCCCTCGCATCCA |  |  |  |
| *Zpu1*_5'F | CCCAAAGGGTGCGTCCCGTC | 146 | 501 | 356 |
| *Zpu1*_5'R | GGCAACATCAACGGAGCTCGGAA |  |  |  |
| *Zpu1*_2F | GCCCAAAGGGTGCGTCCCGT | 185 | 1000 | 816 |
| *Zpu1*_2R | GCCGCCAATCCATCCCAGGCA |  |  |  |
| *Zpu1*_3F | TCGCCTACACTGGACCGCTTG | 642 | 1365 | 724 |
| *Zpu1*_3R | GGAACGCCCCAAACCACAGGG |  |  |  |
| *Zpu1*_4F | GCCTGGGATGGATTGGCGGCT | 980 | 2040 | 1061 |
| *Zpu1*_4R | TGGTCAGCGTATGTAGCAAGCGT |  |  |  |
| *Zpu1*_5F | ACCCTGTGGTTTGGGGCGTT | 1344 | 2464 | 1121 |
| *Zpu1*_5R | GCTCCCTTCGTTCTTTTCTCTTGGTGG |  |  |  |
| *Zpu1*_6F | CAGTTGGCTATGCTTCATCCCCT | 2142 | 2904 | 763 |
| *Zpu1*_6R | GCTGCTGTCCTTTTCGGCACG |  |  |  |
| *Zpu1*_3'F | CACAGGGCCCTCCTTGGTTCC | 2632 | 2965 | 334 |
| *Zpu1*_3'R | TGCCGAATGCACTTGCTCGCT |  |  |  |

^a^ The primer *Zpu1*_1F starts from 5’ promoter region
